# Supplementary material for: Thermo-reversible gelation of self-assembled conducting polymer colloids
Source: Nat Commun. 2025 Dec 5;16:10879. doi: 10.1038/s41467-025-66034-x (PMC12680621; doi:10.1038/s41467-025-66034-x)
Supplement: Supplementary file 1 — Supplementary Information [file 41467_2025_66034_MOESM1_ESM.pdf]

Supplementary information for

Thermo-reversible gelation of self-assembled conducting polymer colloids

Vidhika S. Damani,[1] Xinran Xie,[2] Rachel E. Daso,[2] Khushboo Suman,[3,4] Masoud Ghasemi,[5] Weiran Xie,[1] Ruiheng Wu,[2] Yuhang Wu,[1] Calvin L. Chao,[6] Julian E. Alberto,[3] Casey M. Lorch,[7] Ai-Nin Yang,[8] Dan My Nguyen,[8] Tulaja Shrestha,[8] Kayla Otero,[8] Chun-Yuan Lo,[8] Darrin J. Pochan,[1] Enrique D. Gomez,[5] Jonathan Rivnay,[2,9] Laure V. Kayser[1, 8]*

[1]*Department of Materials Science and Engineering, University of Delaware, Newark, DE 19716, USA*

[2]*Department of Biomedical Engineering, Northwestern University, Evanston, IL 60208, USA*

[3]*Department of Chemical and Biomolecular Engineering, University of Delaware, Newark, DE 19716, USA*

[4]*Department of Chemical Engineering, Indian Institute of Technology Madras, Chennai 600036, India*

[5]*Department of Chemical Engineering and Department of Materials Science and Engineering, the Pennsylvania State University, University Park, PA 16802, USA*

[6]*Department of Surgery, Northwestern University Feinberg School of Medicine, Chicago, IL, 60611, USA*

[7]*Department of Biomedical Engineering, University of Delaware, Newark, DE 19716, USA*

[8]*Department of Chemistry and Biochemistry, University of Delaware, Newark, DE 19716, USA*

[9]*Department of Materials Science and Engineering, Northwestern University, Evanston, IL 60208, USA*

*Corresponding author. Email: lkayser@udel.edu

SI Table of Contents

**1 Materials Synthesis and preparation**	S5
**1.1** Synthesis of α-Methyl trithiocarbonate-S-phenylacetic Acid	S5
**1.2** Synthesis of polystyrene sulfonate chain transfer agent (PSS CTA)	S5
**Fig. S1** Synthesis of PSS macro-CTA	S5
**1.3** Synthesis of PSS-*block*-PNIPAM	S6
**Fig. S2.** Synthesis of PSS-*block*-PNIPAM	S6
**1.4** Synthesis of PEDOT:PSS-*block*-PNIPAM (TR-CP)	S6
**Fig. S3.** Synthesis of PEDOT:PSS-*block*-PNIPAM (TR-CP)	S7
**1.5** Synthesis of PSS-*co*-PNIPAM by RAFT polymerization	S7
**Fig. S4.** Synthesis of PSS-*co*-PNIPAM by RAFT polymerization.	S7
**1.6** Synthesis of PEDOT:PSS-co-PNIPAM	S7
**Fig. S5.** Synthesis of PEDOT:PSS-*co*-PNIPAM.	S8
**1.7** Preparation of TR-CPs with variable concentration (pH = 4).	S8
**1.8** Preparation of TR-CPs with variable concentration (pH = 7).	S8
**1.9** Preparation of the TR-CP solution at basic pH.	S8
**1.10** Preparation of alginate gels	S8
**1.11** Preparation of TR-CP/alginate composites.	S8
**2 Supplementary figures and tables**	S9
**2.1** Molecular characterization of the polymers	S9
**Fig. S6.** GPC of the PSS macro-CTA and PSS-block-PNIPAM block copolymers (BCP)	S9
**Fig. S7a.** ^{1}H NMR spectrum of PSS_{95}-*b*-$PNIPAM_{128}$ in D_2O.	S10
**Fig. S7b**. ^{1}H NMR spectrum of PSS_{176}-*b*-$PNIPAM_{268}$ in D_2O.	S11
**Fig. S7c**. ^{1}H NMR spectrum of PSS_{176}-*b*-$PNIPAM_{494}$ in D_2O.	S12
**Fig. S7d**. ^{1}H NMR spectrum of PSS_{96}-*b*-$PNIPAM_{440}$ in D_2O.	S13
Experimental design of the PSS-*b*-PNIPAM block copolymer for reversible gelation	S14
**2.2** Rheological characterization of the TR-CPs	S15
**Fig. S8.** Rheological characterization of PEDOT:PSS-*block*-PNIPAM.	S15
**Fig. S9.** Additional rheological characterization for PEDOT:PSS_{96}-*b*-$PNIPAM_{440}$ (TR-CP) at 3.6 wt% in water.	S16
**Fig. S10.** Effect of TR-CP concentration in DI water (pH = 7).	S16
**Fig. S11.** Stability of the TR-CP at 37 °C for 80 days.	S17

**2.3** Electronic characterization of TR-CPs	S17
**Fig. S12.** Electronic properties of the TR-CP in the liquid (23 °C) and gel (37 °C) states, 3.6 wt% in DI water.	S17
**Fig. S13.** Electronic properties of TR-CP gel (37 °C, pH = 4) at different concentrations in phosphate buffered saline (PBS).	S18
**Table S1:** Fitting elements for the TR-CP in its liquid and gel states by EIS, 3.6 wt% in DI water.	S18
**Table S2:** Fitting elements for TR-CP at different concentration by EIS, pH = 4 in PBS.	S18
**Table S3.** Conductivity of TR-CPs, pH = 4 in PBS.	S19
Decrease in conductivity on increasing concentration above 5.6 wt%.	S19
**2.4** Characterization of the TR-CP microstructure by cryo-EM	S19
**Fig. S14.** Rheology on 1.8 wt% TR-CP.	S19
**Fig. S15.** Cryo-EM images for 1.8 wt% TR-CP.	S20
**Fig S16.** Analysis of cryo-EM images for 3.6 wt%, below LCST.	S21
**Fig S17.** Fast Fourier Transform (FFT) of cryo-EM images for TR-CP.	S22
**Fig S18.** Cryo-EM micrographs of 1.8 wt% TR-CP at three different defocus values	S23
**Fig S19.** Analysis of cryo-EM images for 1.8 wt% TR-CP, above LCST.	S24
Notes on cryo-EM	S24
Effect of concentration on gelation mechanism for 3.6 wt% vs 5.6 wt% TR-CP.	S25
**Fig S20.** Change in storage modulus as a function of time at 37 °C, for 3.6 and 5.6 wt% TR-CP	S25
**2.5** Characterization of the TR-CP by small-angle X-ray scattering (SAXS).	S26
**Fig. S21.** Small-angle x-ray scattering results on TR-CPs showing measurement, fitting lines and total fit, and raw data from the background.	S27
**Table S4.** Fitting Parameters from SAXS.	S28
**2.6** X-ray photoelectron spectroscopy.	S28
**Fig S22.** Effect of increasing PEDOT:PSS ratio on N:S ratio obtained by XPS on PEDOT:PSS_{96}-*b*-$PNIPAM_{440}$ TR-CPs with variable PSS:PEDOT ratio.	S28
**2.7** In-situ Raman spectroscopy.	S28
**Fig S23.** Variable-temperature Raman spectroscopy on 5.6 wt% PEDOT:PSS_{96}-*b*-$PNIPAM_{440}$ TR-CP.	S28
**Table S5.** Analysis of variable-temperature Raman Spectroscopy.	S29
**2.8** Electronic characterization of the TR-CPs at pH = 7 in PBS	S30
**Fig S24.** Electronic properties of TR-CP gel (37 °C) at different concentrations at pH 7 in PBS.	S30

**Table S6.** Fitting elements for the TR-CP at pH 7 in varying concentration in PBS by EIS.	S30
**Table S7.** Conductivity of TR-CPs at pH = 7 in PBS.	S31
**2.9** *In vitro* cytocompatibility	S31
**Fig. S25.** Preparation of TR-CPs for cell viability experiments.	S31
**Fig S26.** Effect of basic pH on the TR-CP at pH = 9 (top) and pH = 12 (bottom).	S31
**2.9.1** Indirect Extract Tests.	S32
**Fig S27.** Indirect contact assay for 3.6 wt% TR-CP.	S32
**2.9.2** Direct Contact Tests.	S32
**Fig. S28.** Positive control for TR-CP and TR-CP/alginate.	S32
**Fig S29.** Cell viability for 5.6 wt% PEDOT:PSS_{96}-*b*-$PNIPAM_{440}$ TR-CP/alginate.	S33
**2.10** Characterization of Alginate and TR-CP/Alginate composite gels	S33
**2.10.1** Rheological characterization.	S33
**Fig S30.** Rheological characterization of the alginate and 5.6 wt% TR-CP/alginate composite gels.	S33
**2.10.2** Electronic characterization.	S34
**Fig S31.** EIS of alginate and 5.6 wt% TR-CP/alginate gels.	S34
**2.11** In vivo cytocompatibility	S34
Analysis of in vivo cytocompatibility data	S34
**Table S8.** Description of samples and controls for cytocompatibility tests	S35
**2.12** Processing of the TR-CPs	S35
**Fig. S32.** Spreading of TR-CP in cooled viscous liquid.	S35
**3 References**	S35

1 MATERIALS SYNTHESIS AND PREPARATION

1.1 Synthesis of α-Methyl trithiocarbonate-S-phenylacetic Acid (Chain transfer agent, CTA). α-Methyl trithiocarbonate-S-phenylacetic Acid was synthesized as reported by Yusa et. al.[1] 1.08mL of carbon disulfide was dissolved in 9.75mL of diethyl ether. Separately, 1.035g of sodium thiomethoxide was mixed with 30mL diethyl ether. The solution of carbon disulfide was added dropwise to the suspension of sodium methoxide in diethyl ether at room temperature and stirred for 2 hours. Diethyl ether was removed by decantation and evaporation to yield a yellow powder. 33mL of ethyl acetate was added to the yellow powder to dissolve it. The insoluble part was filtered out. To the solution, 2.94g of α-Bromophenylacetic acid was added. The reaction mixture was heated to 70 ℃ and stirred overnight with reflux. The reaction mixture was washed thrice with 1M HCl and subsequently with saturated NaCl solution. The organic layer and aqueous layers were separated using a separating funnel. The organic layer was dried over sodium sulfate and recrystallized from n-hexane/ethyl acetate to obtain α-Methyl trithiocarbonate-S-phenylacetic Acid or MTPA (yield = 600 mg).

1.2 Synthesis of poly(styrene sulfonate) (PSS) macro-chain transfer agent (macro-CTA). PSS was synthesized using sodium styrene sulfonate (NaSS) and α-methyl trithiocarbonate-S-phenylacetic Acid (MTPA) as the CTA for the reversible addition-fragmentation chain transfer (RAFT) polymerization. To obtain PSS with a molecular weight 20 kg mol^{-1}, 2.06g (10 mmol) of NaSS was dissolved in 10 mL water, along with 26 mg (0.1 mmol) of MTPA and 5.6mg (0.02 mmol) of ACVA. The reaction was degassed for 30 minutes under nitrogen. The reaction proceeded for 8 hours at 70 °C to obtain PSS chain transfer agent (**Fig. S1**), which was characterized using NMR and GPC (M_n = 19.9 kg mol^{-1}, yield = 1.97g, $Đ$ = 1.27).

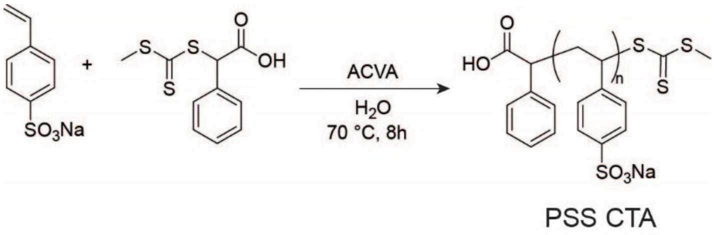

Fig. S1. Synthesis of the PSS macro-CTA by RAFT polymerization.

1.3 Synthesis of PSS-*block*-PNIPAM. PSS-*block*-PNIPAM was synthesized by RAFT polymerization as shown previously by Mizusaki et. al.[2] PSS chain transfer agent was used to synthesize PSS-*block*-PNIPAM. A mixture of water/methanol (1:1 v/v) was used to control the thermo-response of the block copolymer as it was synthesized. 2.26g (20mmol) of NIPAM along with 0.963g (0.05 mmol) of PSS chain-transfer agent and ACVA (5.75mg, 0.02 mmol) were dissolved in 4 ml of 1:1 water-methanol solution. The reaction mixture was degassed for 30 mins under nitrogen. Then, the reaction mixture was heated to 70 °C for 90 minutes or till the reaction formed a viscous gel-like mass, to yield PSS-*block*-PNIPAM (**Fig. S2**). PSS-*b*-PNIPAM was purified by dialysis and characterized for block ratio and molecular weight using NMR. Gel permeation chromatography was used to confirm the formation of block copolymer and obtain dispersity (M_n = 69.6 kg mol^{-1}, yield = 1.8g, $Đ$ = 1.2).

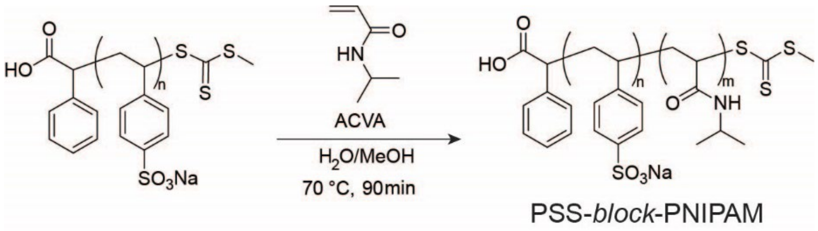

Fig. S2. Synthesis of PSS-*block*-PNIPAM.

1.4 Synthesis of PEDOT:PSS-*block*-PNIPAM (TR-CP). PSS-*b*-PNIPAM was used as a matrix to synthesize thermo-responsive PEDOT:PSS-*b*-PNIPAM dispersions by oxidative polymerization of EDOT. First, PSS-*b*-PNIPAM was stirred over acidic resin (Dowex Marathon C) to acidify the PSS, then dried overnight in an oven. 250 mg of acidified PSS-*b*-PNIPAM was dissolved in 7mL of water. Once completely dissolved, iron (III) chloride (3.5 µL of 10% w/v solution) and sodium persulfate (36.8 mg) were added. After stirring for 10 minutes, 12 µL (0.112 mol) of EDOT was added and the reaction proceeded for 13 h at room temperature to yield PEDOT:PSS-*b*-PNIPAM (TR-CP) (**Fig. S3**). PEDOT:PSS-*b*-PNIPAM was purified by simultaneously stirring over 3mL of acidic resin (Dowex Marathon C) and 2mL basic (Lewatit Ion Exchange) resin.

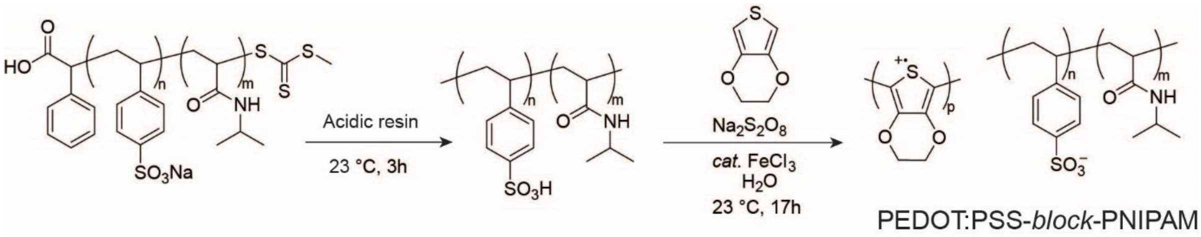

Fig. S3. Synthesis of PEDOT:PSS-*block*-PNIPAM (TR-CP).

1.5 Synthesis of PSS-*co*-PNIPAM by RAFT polymerization. PSS-*co*-PNIPAM was synthesized using α-Methyl trithiocarbonate-S-phenylacetic Acid (MTPA) as the RAFT agent. 2.26g (20mmol) of NIPAM along with 0.963g (4.67 mmol) of NaSS and ACVA (5.75mg, 0.02 mmol) were dissolved in 4 ml of 1:1 water-methanol solution. The reaction mixture was degassed for 30 mins under nitrogen and the heated to 50 °C till NIPAM was completely dissolved. Then, the reaction mixture was heated to 70°C for 16 hours to yield PSS-*co*-PNIPAM (**Fig. S4**) (M_n = 70 kg mol^{-1}, yield = 2.1 g, $Đ$ = 1.38).

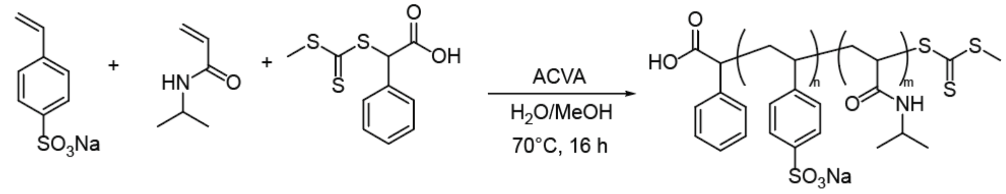

Fig. S4. Synthesis of PSS-*co*-PNIPAM by RAFT polymerization.

1.6 Synthesis of PEDOT:PSS-*co*-PNIPAM. PSS-*co*-PNIPAM was used as a matrix to synthesize thermo-responsive PEDOT. PEDOT:PSS-*co*-PNIPAM was synthesized by oxidative polymerization. First, PSS-*co*-PNIPAM was stirred over acidic resin (Dowex Marathon C) to acidify the PSS. PSS-*co*-PNIPAM was dried overnight in an oven. 250 mg of acidified PSS-*co*-PNIPAM was dissolved in 7mL of water. Once completely dissolved, iron (III) chloride (3.5 μL of 10% w/v solution) and sodium persulfate (36.8 mg) were added. After stirring for 10 minutes, EDOT (12 μL, 0.112 mol) was added and the reaction proceeded for 13 hours at room temperature to obtain PEDOT:PSS-*co*-PNIPAM (**Fig. S5**). PEDOT:PSS-*co*-PNIPAM was purified by stirring simultaneously over 3mL of acidic and 2mL basic resins.

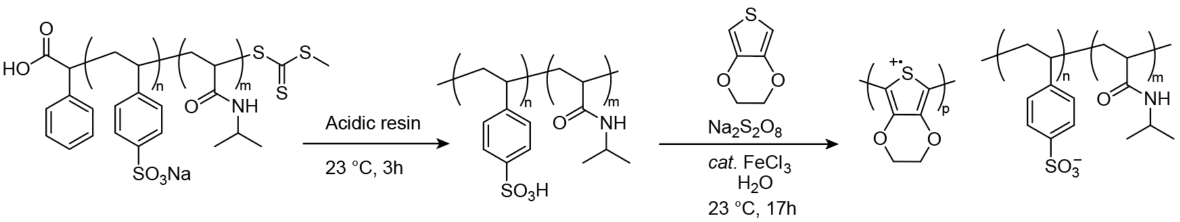

Fig. S5. Synthesis of PEDOT:PSS-*co*-PNIPAM.

1.7 Preparation of TR-CPs with variable concentration (pH = 4). PEDOT:PSS_{96}-*b*-$PNIPAM_{440}$ was lyophilized for 48 hours. The powder thus obtained was dispersed in 1X PBS at different concentrations (3.6 wt%, 5.6 wt% and 7.6 wt%) to yield TR-CPs with variable concentration at pH = 4.

1.8 Preparation of TR-CPs with variable concentration (pH = 7). PEDOT:PSS_{96}-*b*-$PNIPAM_{440}$ (11.1 mL, 3.6 wt% in water) was mixed with 4.1 mL of 0.1M NaOH to achieve a neutral pH of 7-7.4. Then, the dispersion was dialyzed against DI water for 24 hours and lyophilized for 48 hours. The powder thus obtained was dispersed in DI water or 1X PBS at different concentrations (3.6 wt%, 5.6 wt% and 7.6 wt%) to yield TR-CPs with pH = 7.

1.9 Preparation of the TR-CP solution at basic pH. PEDOT:PSS_{96}-*b*-$PNIPAM_{440}$ (TR-CP) was lyophilized and reconstituted in 1X PBS (5.6 wt%). 0.1M NaOH was added dropwise using a micropipette to adjust the pH to 9 and 12 without drastically changing the concentration. Additionally, to make a sample with pH = 14, 56 mg of the TR-CP was added to 1mL of 1M NaOH, and we attempted to disperse the TR-CP by stirring overnight.

1.10 Preparation of alginate gels. Sodium alginate (100 mg) was added to 10mL DI water to yield 1 wt% sodium alginate solution. The solution was sonicated for 4 hours and then stirred overnight. Obtained sodium alginate solution was poured onto a glass petri dish and 20 mM barium chloride solution was poured gently on top. The gel was covered with a glass slide, weighed down and allowed to gel for 2 hours. After the gelation was complete, the alginate gel was washed with DI water and stored in a closed petri dish.

1.11 Preparation of TR-CP/alginate composites. Sodium alginate (100 mg) was added to 10mL DI water to yield 1 wt% sodium alginate solution. Then, neutralized TR-CP powder (56 mg) was added to 1 mL of the solution in a vial. The mixture was sonicated for

4 hours and then stirred overnight. This blend was poured onto a glass petri dish and 20 mM barium chloride solution was poured gently on top. The gel was covered with a glass cover slip and allowed to gel for 2 hours. After the gelation was complete, obtained TR-CP/alginate composite was washed with DI water and stored in a closed petri dish.

2 SUPPLEMENTARY FIGURES AND TABLES

2.1 Molecular characterization of the polymers

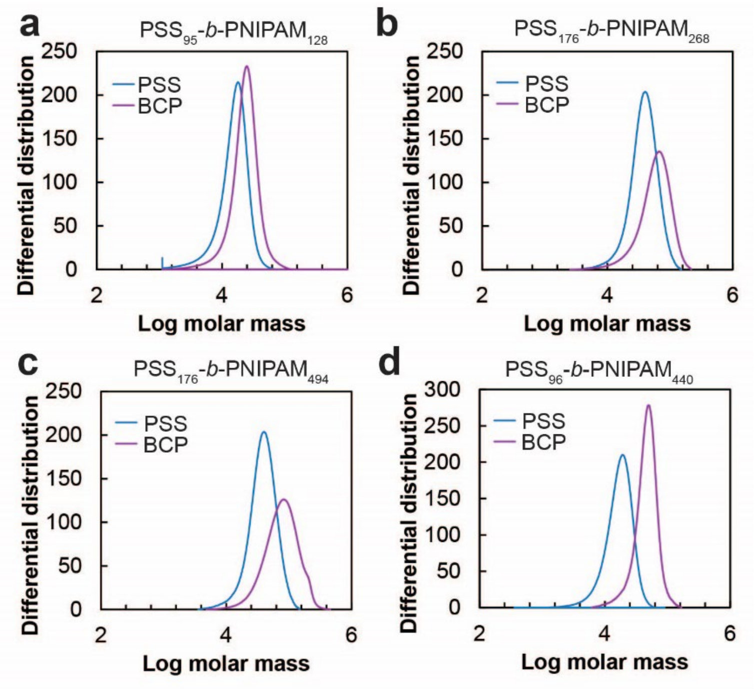

Fig. S6. GPC of the PSS macro-CTA and PSS-*block*-PNIPAM block copolymers (BCP) for **a.** PSS_{95}-*b*-$PNIPAM_{128}$, **b.** PSS_{176}-*b*-$PNIPAM_{268}$, **c.** PSS_{176}-*b*-$PNIPAM_{494}$, **d.** PSS_{96}-*b*-$PNIPAM_{440}$.

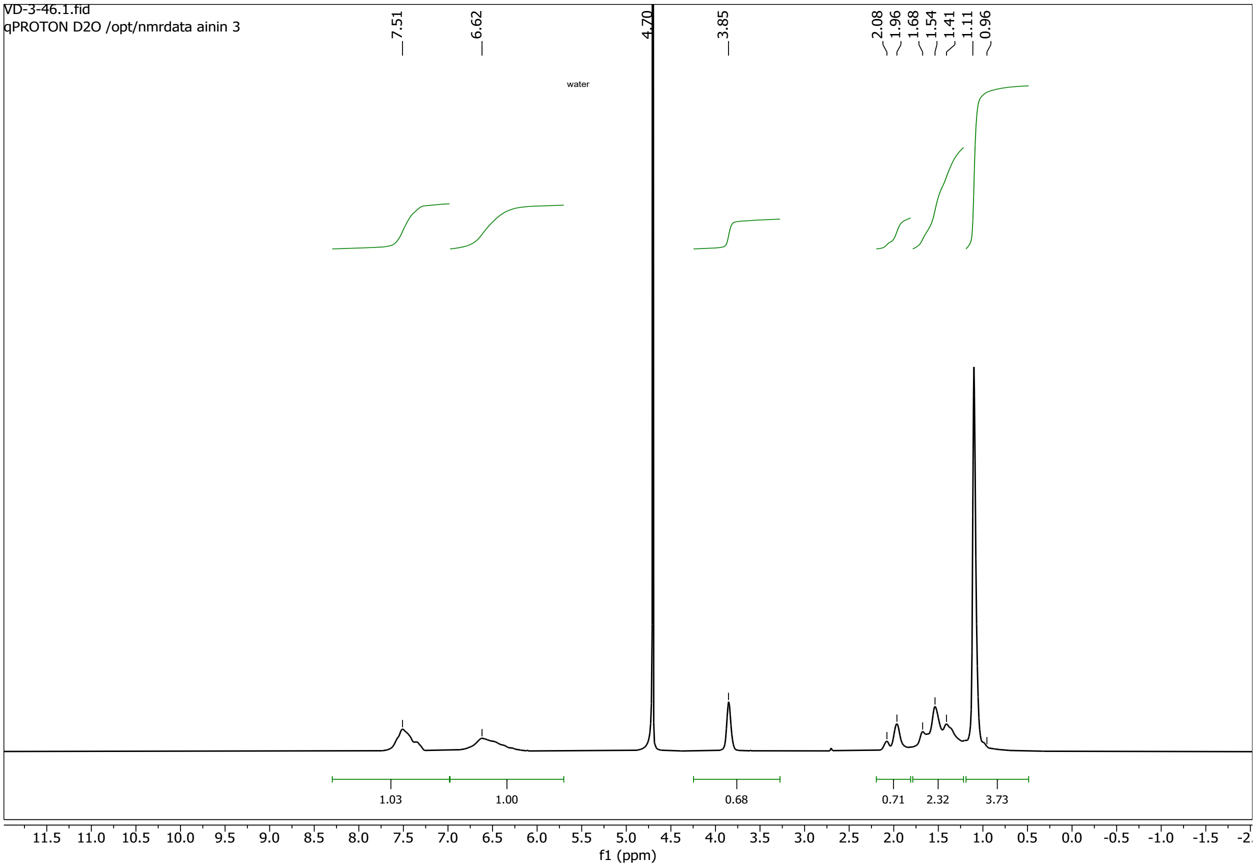

Fig. S7a. ^{1}H NMR spectrum (400 MHz, ambient temperature) of PSS_{95}-*b*-$PNIPAM_{128}$ in D_2O.

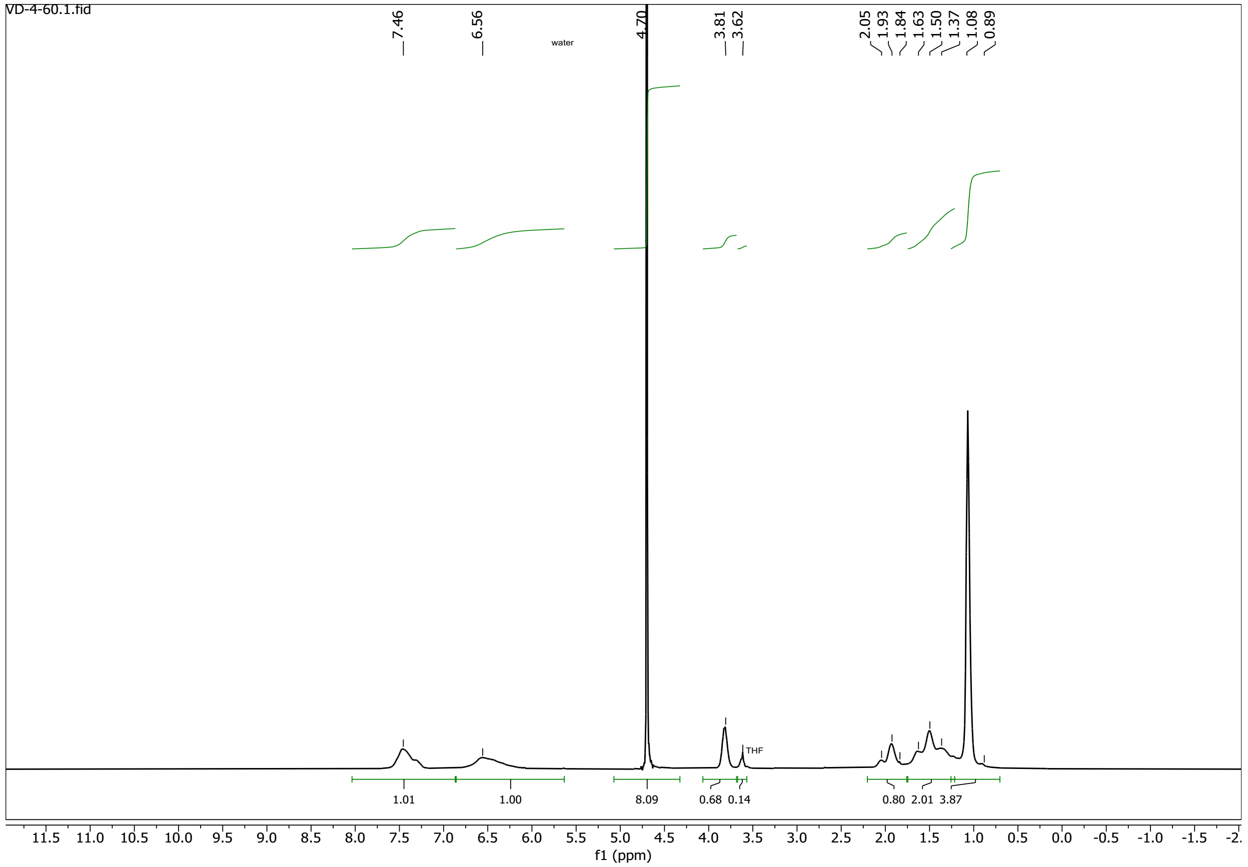

Fig. S7b. ^{1}H NMR spectrum (400 MHz, ambient temperature) of PSS_{176}-*b*-$PNIPAM_{268}$ in D_2O.

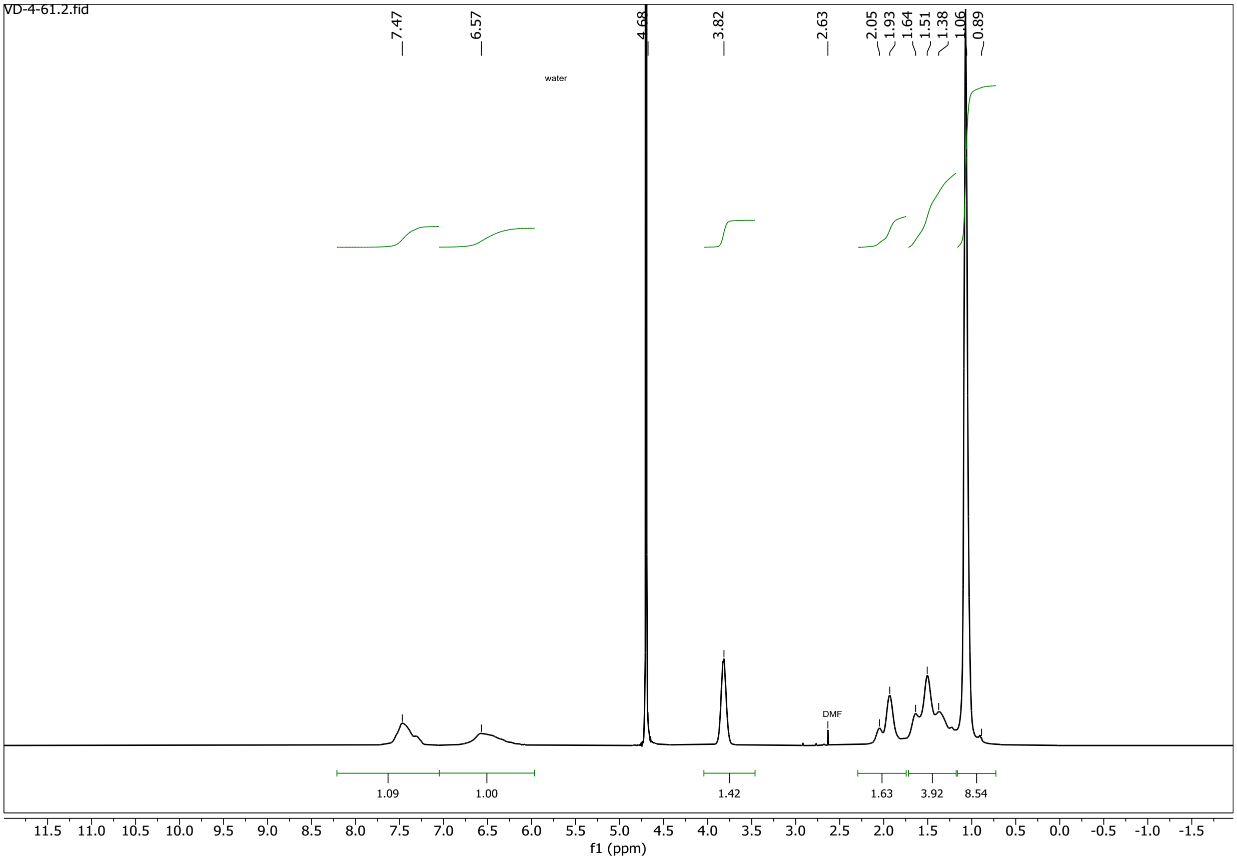

Fig. S7c. ^{1}H NMR spectrum (400 MHz, ambient temperature) of PSS_{176}-*b*-$PNIPAM_{494}$ in D_2O.

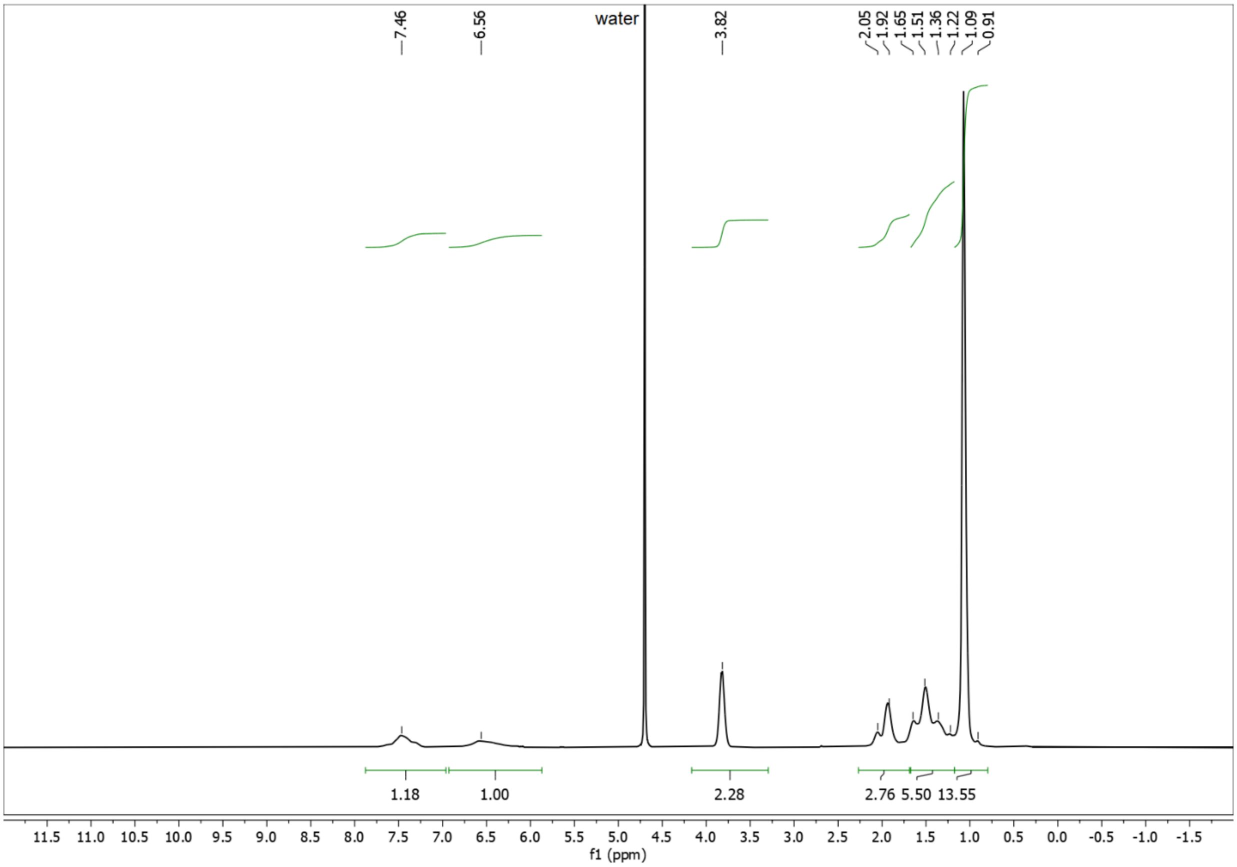

Fig. S7d. ^{1}H NMR spectrum (400 MHz, ambient temperature) of PSS_{96}-*b*-$PNIPAM_{440}$ in D_2O.

Experimental design of the PSS-*b*-PNIPAM block copolymer for reversible gelation. TR-CPs with varying molecular weight and ratio of PSS: PNIPAM were synthesized using RAFT polymerization. To observe the thermo-response, we recorded the storage modulus (G') and loss modulus (G") as a function of temperature. Trends in G' and G" can be used to understand the molecular mechanism of thermo-response, and its effect on gelation (or lack thereof).[3] Typically, for liquids, G' is either lower than or close to G". As the system reaches closer to a gelation, the G' crosses over G", demonstrating the ability of the material to display primarily elastic behavior, which is indicative of gelation. We used this G'– G" crossover to determine the presence of a sol-gel transition.[4] The angular frequency for all these measurements was kept the same (10 rad s^{-1}) to compare the samples under similar shear, and was chosen based on the stability of the dispersions under these conditions. The types of thermo-response (increase in viscosity, formation of a weak gel, etc.) recorded from rheology were in good agreement with our visual observations.

The TR-CP with PSS:PNIPAM 1:0.73 by mass (Table 1, Entry 1) showed an increase in both G' and G" at 37 °C, but no crossover. This result indicated that PEDOT:PSS_{95}-*b*-$PNIPAM_{128}$ displayed a slight increase in modulus, but no gelation at least up to 50 °C (**Fig. S8 a-b**). The same trend was observed on recording G' and G" at 37 °C. The lack of visible thermo-response was attributed to the low overall molecular weight of the block copolymer, as the thermo-response was likely not strong enough to induce gelation By doubling the molecular weight of both blocks **(**Table 1, Entry 2), an increase in modulus was observed at 37 °C, but gelation only happened at 38 °C (**Fig. S8c-d**). Increasing the overall molecular weight indeed induced a stronger thermo-response (as seen by the presence of a crossover point in **Fig. S8c**, and by the higher G' and G" of PEDOT:PSS_{176}-*b*-$PNIPAM_{268}$ as compared to PEDOT:PSS_{95}-*b*-$PNIPAM_{128}$). However, since the gelation occurred above physiological temperatures, we deemed this sample unsuitable for applications in bioelectronics. Thus, to lower the gelation temperature, the molecular weight of the PNIPAM block was increased while maintaining the same PSS block size (**Table 1, Entry 3**). This TR-CP gelled at 36 °C, however, the gel was not stable at 37 °C for prolonged times and at volumes larger than 1 mL (**Fig. S8e-f**). The time sweep at 37 °C displayed a steep increase in both G' and G", but no crossover was observed, which indicated the formation of a weak gel. The unstable gelation could be attributed to the low percentage of PNIPAM, and subsequently, weak thermo-response in the gel. To address this issue, and to further lower the gelation temperature by strengthening the thermo-response, the PNIPAM block was kept 50 kg mol^{-1}, but we decreased

the molecular weight of PSS to 18 kg mol^{-1} (**Table 1, Entry 4)**. This TR-CP with a mass ratio of 1:2.3 PSS:PNIPAM showed the formation of a stable, fully reversible gel at 35 °C, as observed by the G' – G" crossover in the temperature sweep and time sweep.

2.2 Rheological characterization of the TR-CPs

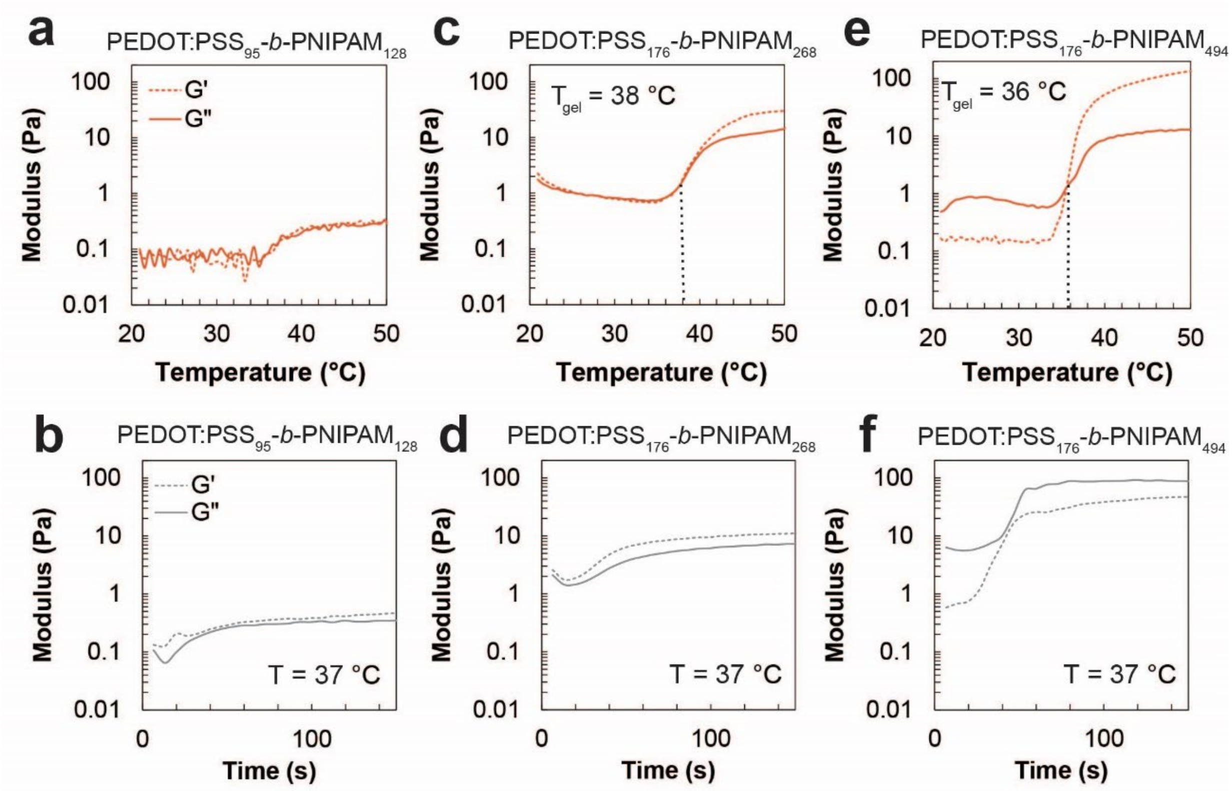

Fig S8. Rheological characterization of PEDOT:PSS-*block*-PNIPAM, monitoring the change in loss modulus (G') and storage modulus (G"). **a.** Temperature sweep and **b.** Time sweep at 37 °C for PEDOT:PSS_{95}-*b*-$PNIPAM_{128}$. **c**. Temperature sweep and **d.** Time sweep at 37 °C for PEDOT:PSS_{176}-b-$PNIPAM_{494}$, **e.** Temperature sweep and **f.** Time sweep at 37 °C for PEDOT:PSS_{96}-*b*-$PNIPAM_{440}$.

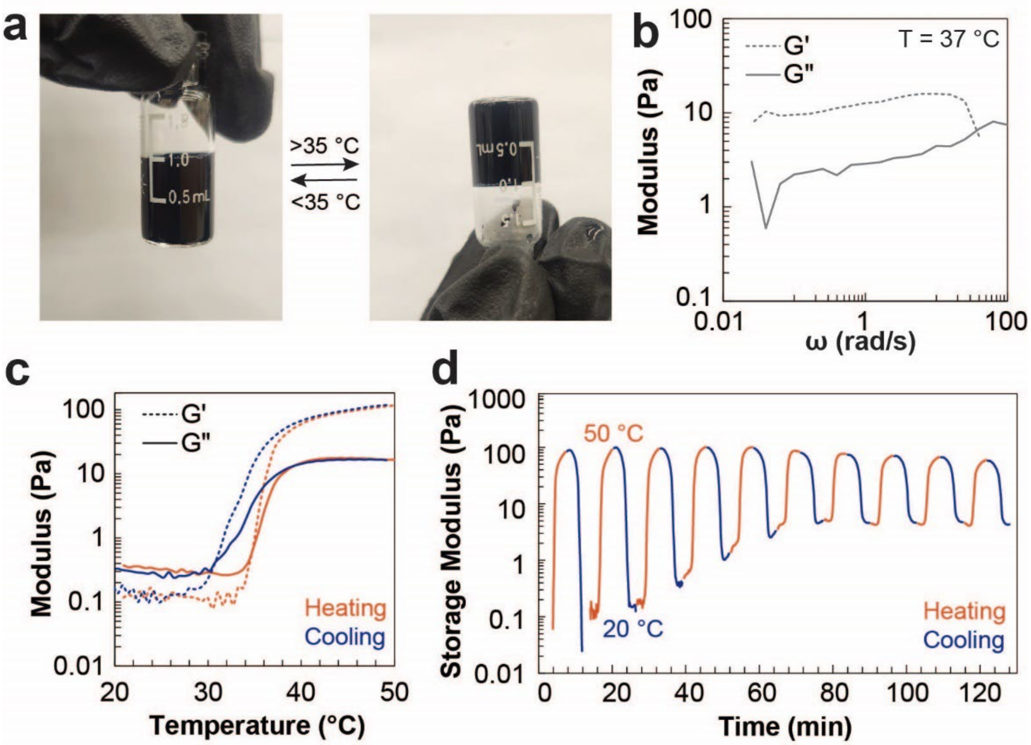

Fig S9. Additional rheological characterization for PEDOT:PSS$_{96}$-*b*-PNIPAM$_{440}$ (TR-CP) at 3.6 wt% in water. **a.** Reversible isovolumetric gelation in TR-CP, observed in a graduated vial. **b.** Change in G’ and G” as a function of angular frequency at 37 °C. **c.** Change in G’ and G” as a function of temperature, monitored during heating and subsequent cooling. **d.** Reversibility of gelation and cycling stability, determined by monitoring the storage modulus (G’) over 10 heat-cool cycles from 20 °C to 50 °C.

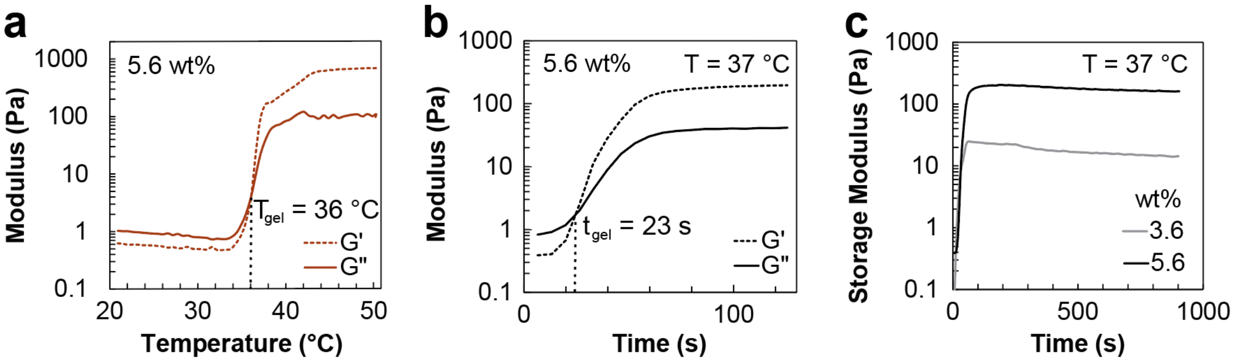

Fig S10. Effect of TR-CP concentration in DI water (pH = 7). **a.** Change in G’ and G” as a function of temperature, upon heating from 20 °C to 50 °C, for TR-CP at 5.6 wt% in DI water. **b.** Change in storage modulus (G’) and loss modulus (G”) as a function of time, at a constant temperature of 37 °C, for TR-CP at 5.6 wt% in water. **c.** Change in G’ as a function of time at 37 °C, for 3.6 wt% and 5.6 wt% TR-CP in water.

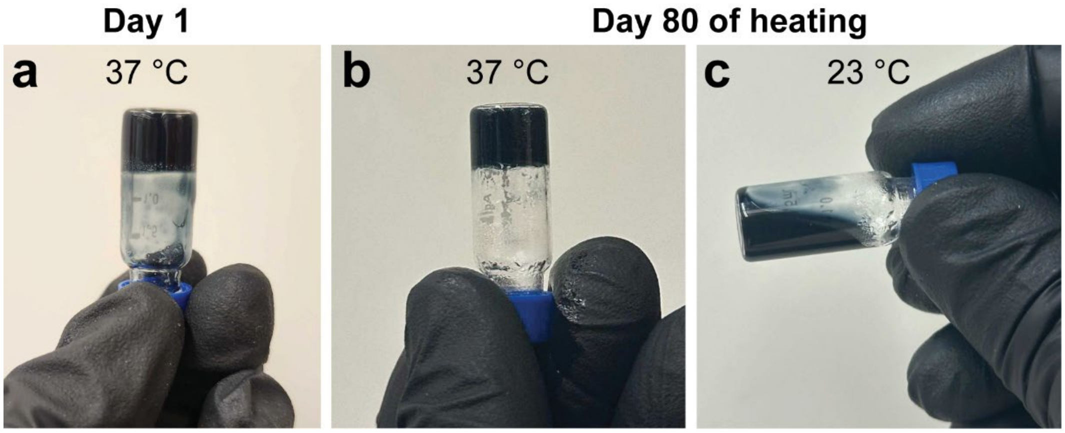

Fig. S11. Stability of the TR-CP gel at 37 °C for 80 days. ~ 0.5 mL of the 5.6 wt% TR-CP in DI water was added to a vial, and heated to 37 °C in an oil bath. The temperature of the bath was maintained at 37 °C for 80 days. a. Picture of the TR-CP gel on Day 1 b. Picture of the TR-CP gel on Day 80, showing negligible changes in volume and stability. c. Picture of the TR-CP reversed to its liquid state on cooling to room temperature (23 °C), after 80 days of heating at 37 °C.

2.3 Electronic characterization of TR-CPs

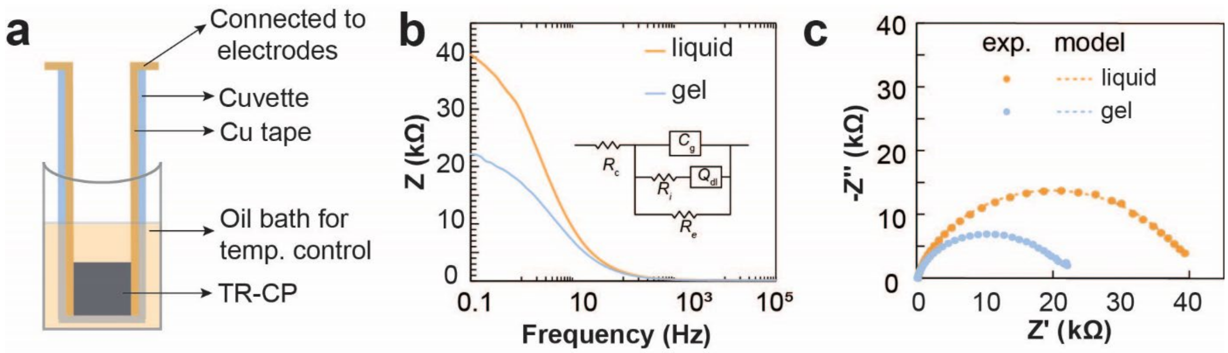

Fig S12. Electronic properties of the TR-CP in the liquid (23 °C) and gel (37 °C) states, 3.6 wt% in DI water. Sample volume = 1 cm^3 **a.** Custom set-up for the electrochemical impedance spectroscopy. **b.** Bode plot for TR-CP liquid and gel, with equivalent circuit model (inset). **c.** Nyquist plot for TR-CP liquid and gel.

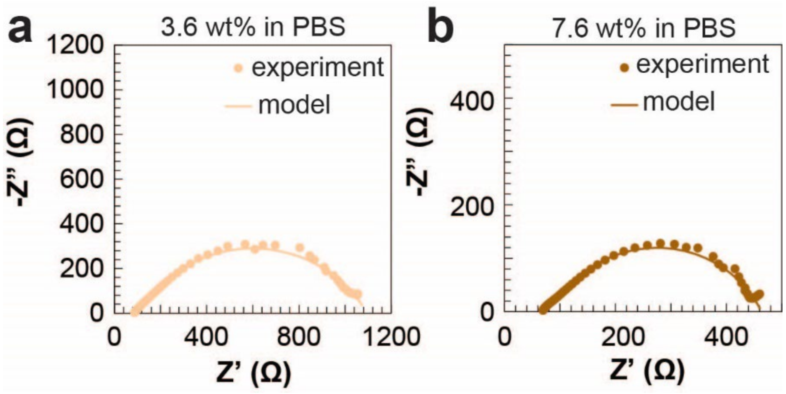

Fig S13. Electronic properties of TR-CP gel (37 °C, pH = 4) at different concentrations in phosphate buffered saline (PBS). Sample volume = 0.4 cm^3 Nyquist plot for TR-CP at **a.** 3.6 wt% in PBS. **b.** 7.6 wt% in PBS.

Table S1. **Fitting elements for the TR-CP in its liquid and gel states by EIS, 3.6 wt% in DI water.**

Elements	Liquid (25 °C)	Gel (37 °C)
R_C (Ω)	194.1	163.2
R_e (kΩ)	41.1	22.5
R_i (Ω)	86.9	121.7
Q_{dl} $(F \cdot S^{N-1})$	3.94×10^{-6}	5.98×10^{-6}
N_{dl}	0.67	0.63
C_g (F)	5.86×10^{-7}	7.08×10^{-7}
χ^2	0.0002	0.0004
Sample volume (cm^3)	1	1
R from 2-point probe (kΩ)	38.1	20.1

Table S2. **Fitting elements for TR-CP at different concentration by EIS, pH = 4 in PBS.**

Elements	3.6 wt% gel	5.6 wt% gel	7.6 wt% gel
R_C (Ω)	89.5	66.7	69.8
R_e (Ω)	1010	232	394
R_i (Ω)	45.9	27.5	31.0
Q_{dl} $(F \cdot S^{N-1})$	5.74×10^{-7}	1.29×10^{-7}	5.94×10^{-7}
N_{dl}	0.66	0.708	0.708
C_g (F)	1.03×10^{-6}	4.07×10^{-6}	1.21×10^{-6}
χ^2	0.04	0.03	0.03
Sample volume (cm^3)	0.4	0.6	0.4
R from 2-point probe (Ω)	1200.0	246.3	450.1

Table S3. Conductivity of TR-CPs, pH = 4 in PBS.

Sample	σ [a] (mS cm^{-1})	σ_i [b] (mS cm^{-1})	σ_e [b] (mS cm^{-1})
3.6 wt% gel	2.08	54.49	2.47
5.6 wt% gel	10.4	126 ± 57*	8.3 ± 1.4*
7.6 wt% gel	5.55	80.64	6.34

[a]electronic conductivity calculated from 2-point probe. [b]ionic and electronic conductivity calculated from EIS. *average of 3 values from independently synthesized samples.

__**Decrease in conductivity on increasing concentration above 5.6 wt%:**__ Visually, we observed that the 7.6 wt% TR-CP did not disperse well as compared to the 3.6 wt% and 5.6 wt% TR-CP, and showed the presence of clumps or aggregates. We believe that these solid clumps hinder the diffusion of ions. Additionally, the gel is not continuous anymore which limits electronic conduction. The non-homogeneity of the sample would also cause disruptions to contact with the electrode.

2.4 Characterization of the TR-CP microstructure by cryo-EM

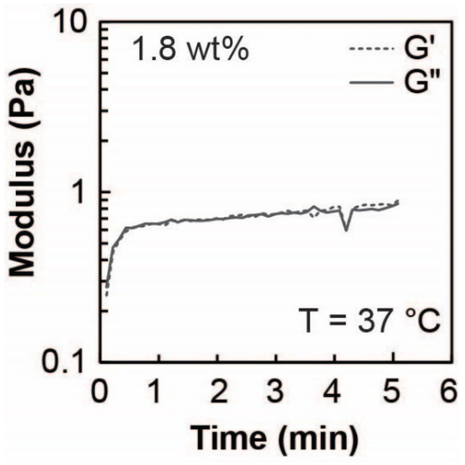

Fig S14. Rheology on 1.8 wt% TR-CP. Change in G' and G" as a function of time at 37 °C shows an increase in modulus, but no gelation.

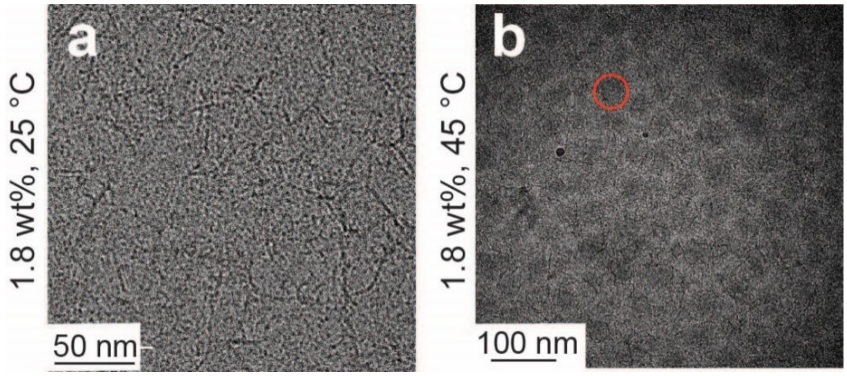

Fig S15. Cryo-EM images for 1.8 wt% TR-CP. **a.** Below LCST. **b.** Above LCST.

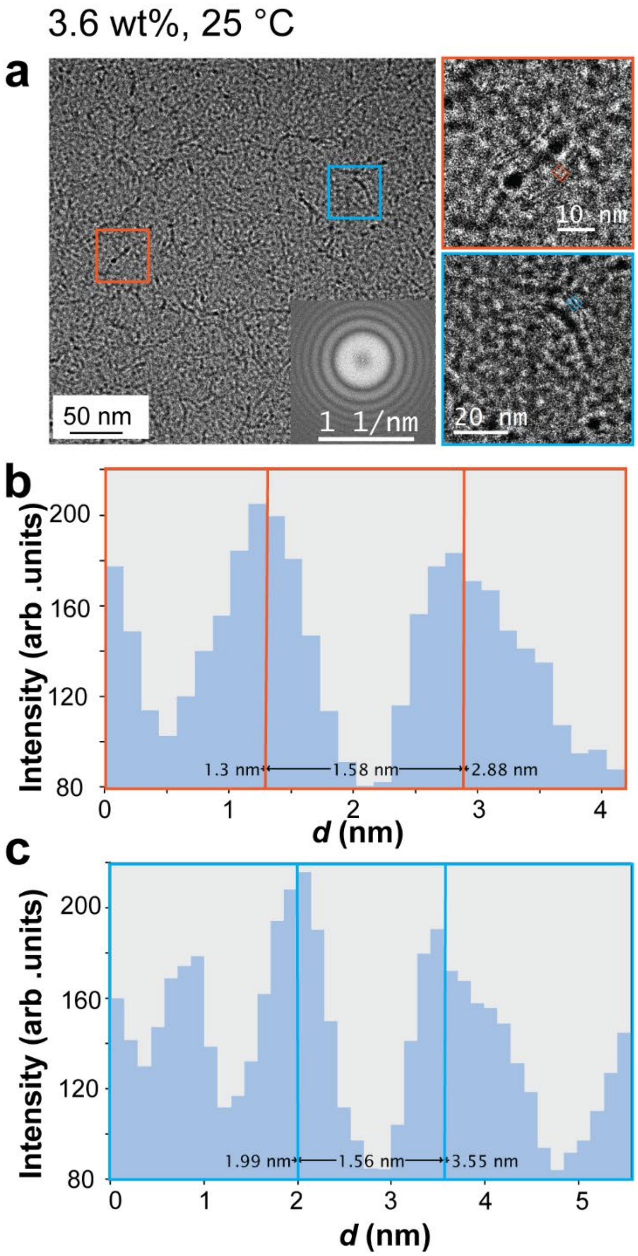

Fig S16. Analysis of cryo-EM images for 3.6 wt%, below LCST. **a.** Cryo-EM image with insets displaying lamellar fringes and FFT. **b, c.** Line plot displaying distance between lamellar fringes; Change in intensity as a function of distance.

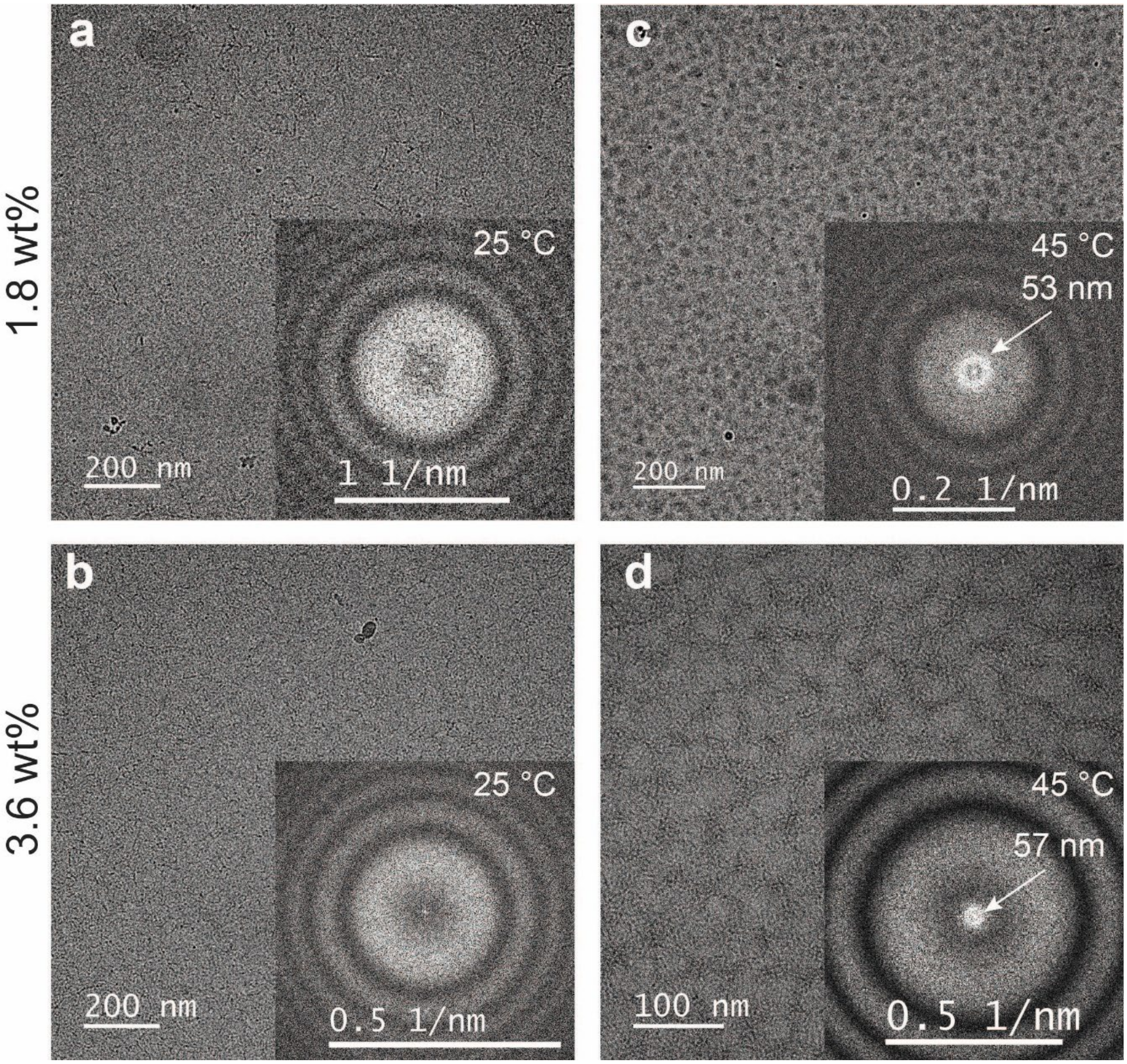

Fig S17. Cryo-EM micrographs and their Fast Fourier Transforms (FFTs), shown as inset, for TR-CP. **a.** 1.8 wt%, below LCST. **b.** 3.6 wt%, below LCST **c.** 1.8 wt%, above LCST showing the formation of spherical structures spaced by 53 nm. **d.** 3.6 wt%, above LCST showing the formation of a bi-continuous network with a mesh size of 57 nm.

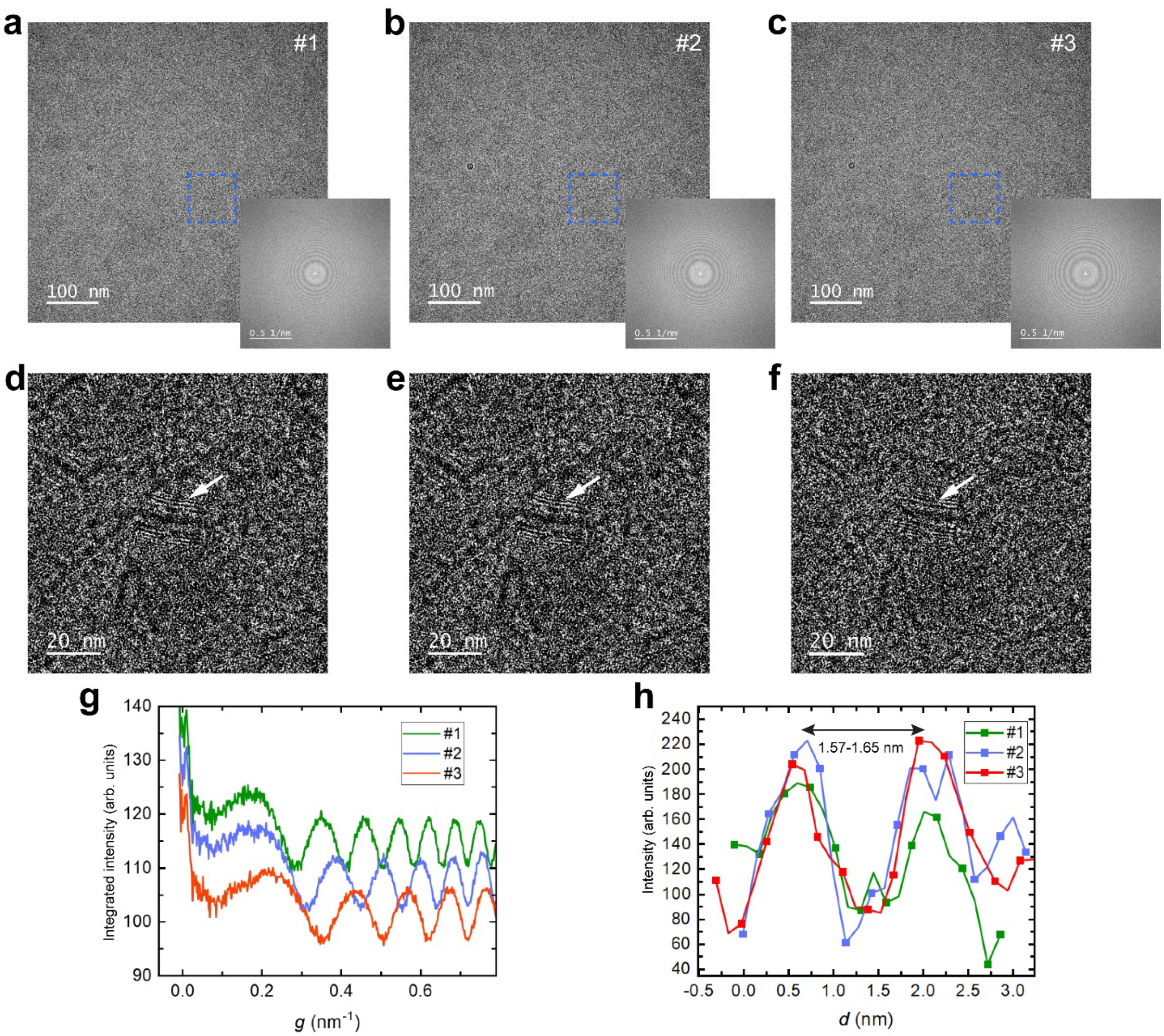

Fig. S18. Cryo-EM micrographs of 1.8 wt% TR-CP, above LCST, at three different defocus values; **a.** large (3.5 µm), **b.** medium (1.9 µm), and **c.** small (1.5 µm) defocus values. EMAN2[5] software was used for contrast transfer function (CTF) fit and defocus calculation. 0.14 nm pixel size, 0.01 mm spherical aberration, and 7% amplitude contrast values were used as initial input for the CTF fits. Insets: 2D FFTs of the corresponding images. Panels **d**, **e**, and **f**, show the zoomed-in view of blue boxes shown in **a**, **b**, and **c**, respectively. White arrows denote lamella fringes observable in micrographs. **g**. Azimuthal integration of FFTs of micrographs shown in panels **a**, **b**, and **c**, as the intensity versus spatial frequency/reciprocal space vector (*g*). The large number of oscillations is from CTF, which varies with defocus value. Azimuthal integration was carried out using Fiji software with the built-in Radial Integration plugin. **h.** Line scans of the lattice fringes in real space as change in intensity versus distance. The peak-to-peak distance shows lamella spacing of PEDOT:PSS. Comparing **g** and **h**, the spacing of the lamella does not depend on defocus, indicating that the spacing corresponds to PEDOT:PSS structure and not an artefact from the oscillations in the CTF. As can be seen in panel **g**, Azimuthal integrated FFT profiles show no distinguishable peak associated with lamella spacing around 0.6 nm^{-1} (1.64 nm), aside from the CTF oscillations. Although FFTs of particular regions of interest, as shown in Fig. S14, show peaks associated with lamella spacing, FFTs of larger images do not exhibit these spacings. This is due to the low density of crystalline domains in these micrographs, and is consistent with the lack of peaks associated with the lamella in solution SAXS results.

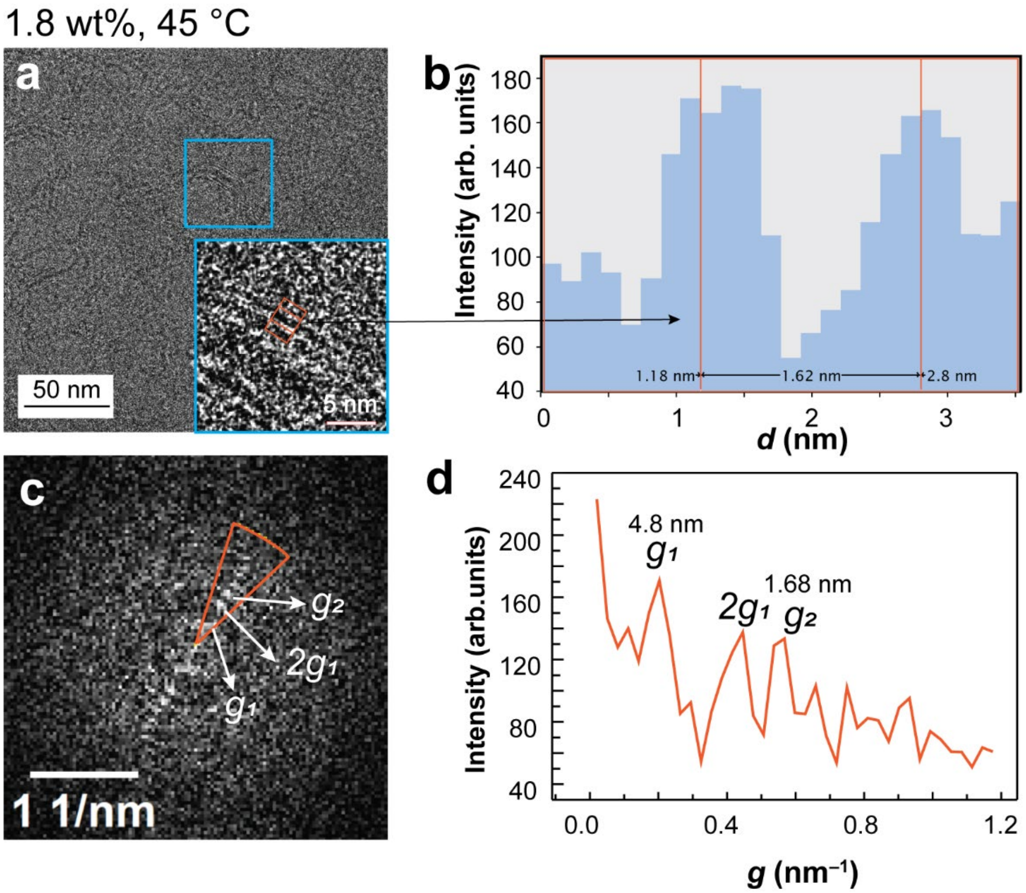

Fig S19. Analysis of cryo-EM images for 1.8 wt% TR-CP, above LCST. **a.** Cryo-EM image with inset displaying lamellar fringes. **b.** Line plot displaying distance between lamellar fringes; Change in intensity as a function of distance. **c.** Fast Fourier Transform of inset image, used to calculate inter-micelle and inter-lamella spacing. **d.** Change in intensity as a function of spatial frequency (*g*); where g_1 is the spacing between micelles ($2g_1$ is the second-order peak of that spacing), and g_2 is the lamella spacing.

Notes on Cryo-EM

The contrast observed in cryo-EM results arise from a combination of phase contrast due to differences in electrostatic (inner) potential between domains, and from differences in mass density, including between crystalline and amorphous regions.[6] Subsequently, because PSS chains and PNIPAM are amorphous, contrast between PSS and PNIPAM domains is minimal. Previous reports show that PEDOT:PSS in films and dispersion phases consist of two different crystalline structures, namely, PEDOT:PSS core-shell micelles with a crystalline PEDOT core,[7] and PEDOT:PSS lamella stacks with alternating PEDOT and PSS chains.[8] In this study, domains containing the latter structures are referred to as crystalline domains, while the core-shell structures are referred to as core-shell micelles. We distinguish between these two different structures, as the

core-shell micelles generally possess a more uniform size distribution, [9] while the crystalline domains in conjugated polymers vary in size and shape within the same samples.[10] Further studies are needed to elucidate the crystalline structure within the PEDOT-rich core of micelles.

The lack of a peak corresponding to PEDOT:PSS lamella spacing in solution SAXS is consistent with prior reports.[7,8] We attribute the lack of lamella features in solution SAXS to the low density of crystalline domains in the solution phase, low number of stacked chains, and overlap with background scattering. On the other hand, in the solid phase, PEDOT:PSS generally shows a higher density of crystalline structures with larger crystalline domains[11] compared to the solution phase. As a result, the scattering signal from these crystalline domains is stronger, which can be readily detected using SAXS/WAXS techniques [12].

Effect of concentration on the gelation mechanism: 3.6 wt% vs 5.6 wt% TR-CP

_A close look at the storage modulus of the 5.6 wt% and 3.6 wt% TR-CP during the time sweep (**Fig. S20**) revealed subtle differences in the heating profiles. While the 3.6 wt% TR-CP showed just one slope (= 0.55), the 5.6 wt% displayed two slopes – one, similar to the 3.6 wt% TR-CP at 0.81, and a second slower heating regime with a much higher slope of 5.18. This difference suggests the occurrence of a 'pre-gel' state in the 5.6 wt% before the formation of a fully interconnected network.

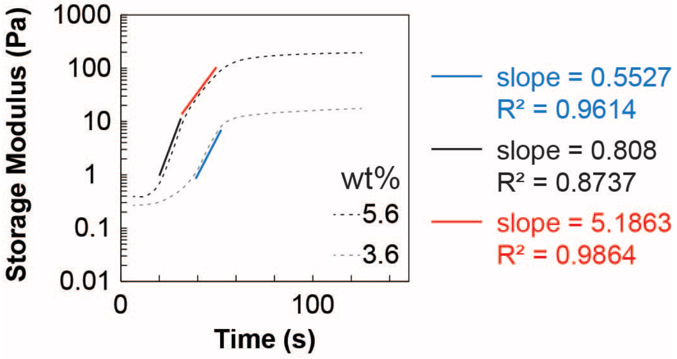

Fig. S20. Change in storage modulus as a function of time at 37 °C, for 3.6 and 5.6 wt% TR-CP. Analysis of the slopes show that the 5.6 wt% TR-CP has two crosslinking 'events' that lead to the formation of a stable gel, which hints at differences in the mechanism of crosslinking for different concentrations.

2.5 Characterization of the TR-CP by small-angle X-ray scattering (SAXS).

We studied the temperature-dependent self-assembly behavior in the TR-CP dispersions using liquid SAXS. The SAXS curves provide information about the polymer at multiple different length scales.[13-15] At low q range (q < 0.2 nm^{-1}), the scattering curve reveals the fractal clusters formed by the polymer aggregates at larger scales. In this work, the SAXS of TR-CP sample above the LCST exhibits a scattering peak centered at 0.102 nm^{-1} (marked as Gaussian Peak 1 in **Fig. S21C**), which corresponds to a characteristic length scale of 61 nm. In the intermediate q range (0.2 < q < 1 nm^{-1}), the curve provides morphological information about the overall dimensions (radius or length) of the aggregates, their shape (spherical, cylindrical, or irregular), and spatial organization within the sample. At higher q values (q > 1 nm^{-1}), the scattering signal reflects the interchain lamellar stacking within the aggregates.

In this work, we fitted the intermediate q features with core-shell sphere model with a hard sphere structure factor (**Table S4**).[16] The core-shell sphere model treated the nanoparticles as spheres with PEDOT core inside and PSS-b- PNIPAM shell outside. The scattering intensity is described by:

$$I(q) = A \cdot F^2(q) \cdot S(q)$$

Where $S(q)$ is the hard sphere structure factor described in[6], A is the scaling factor and $F^2(q)$ is the form factor of core-shell sphere:

$$F(q) = \frac{3}{V_s}\left[V_c \Delta\rho_{c-s} \frac{\sin(qr_c) - qr_c \cos(qr_c)}{(qr_c)^3} + V_s \Delta\rho_{s-w} \frac{\sin(qr_s) - qr_s \cos(qr_s)}{(qr_s)^3}\right]$$

Where V_s is the volume of the whole particle, V_c is the volume of the core, r_s is the radius of the whole particle and is equal to the sum of the core radius r_c and the shell thickness. $\Delta\rho_{c-s}$ and $\Delta\rho_{s-w}$ are the scattering length density difference between core-shell and shell-water, respectively. For the sample below the LCST, the radius of the PEDOT core is 3.2 nm, with a PSS-*b*-PNIPAM shell thickness of 0.9 nm. The average interparticle distance in solution is fitted to be 24 nm, which is twice the fitted effective radius. As the temperature increased, the size of the polymer aggregates (both the core radius and shell thickness) increased (**Table S4**), which may be related to the slight changes in the swelling shell of the polymer at different temperatures. The interparticle distance (effective radius) of the nanospheres decreased above the LCST, consistent with their transition into a gel state through aggregation.

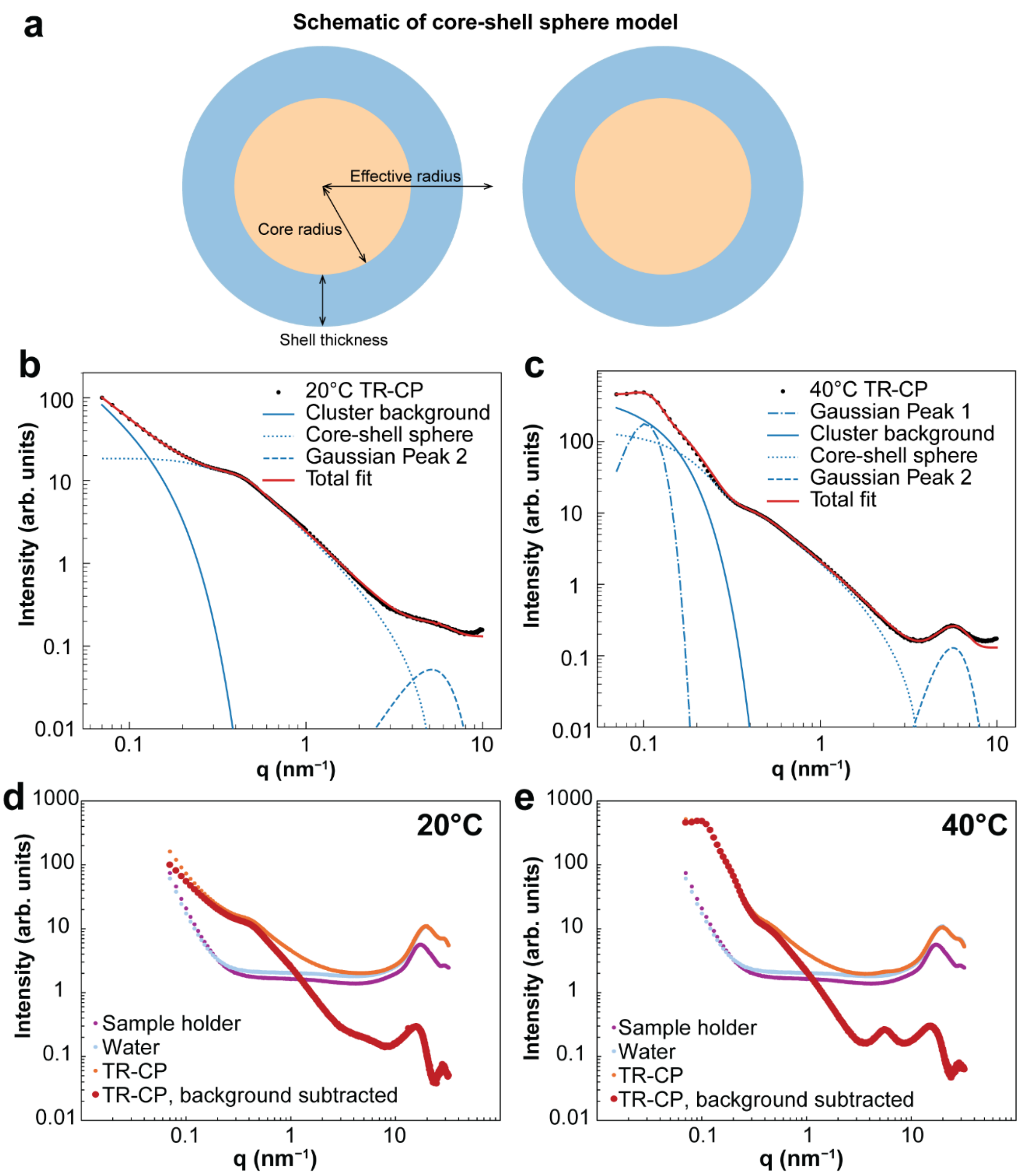

Fig. S21. a. Core shell sphere model on Small-angle x-ray scattering results on TR-CP showing measurement, fitting lines and total fit **b.** below LCST and **c.** above LCST. **d – e.** Raw data from small-angle x-ray scattering for sample holder and water, compared to the TR-CP, **d.** below LCST and **e.** above LCST. 'Background substracted' means that the signals from the sample holder and water were substracted from the TR-CP measurements.

Sample	Core radius (nm)	Shell thickness (nm)	Effective radius (nm)
TR-CP below LCST	3.2 ± 0.05	0.9 ± 0.01	12.2 ± 0.04
TR-CP above LCST	3.9 ± 0.11	1.3 ± 0.01	5.8 ± 0.12

Table S4. Fitting Parameters from SAXS.

2.6 X-ray photoelectron spectroscopy.

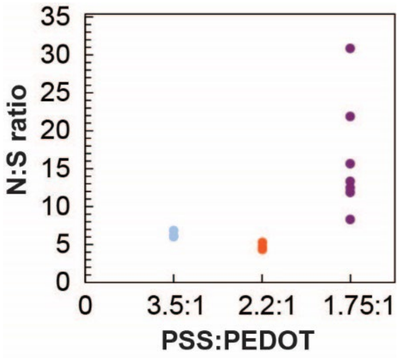

Fig S22. Effect of increasing PEDOT:PSS ratio on N:S ratio obtained by XPS on PEDOT:PSS_{96}-*b*-$PNIPAM_{440}$ TR-CPs with variable PSS:PEDOT ratio.

2.7 In-situ Raman spectroscopy.

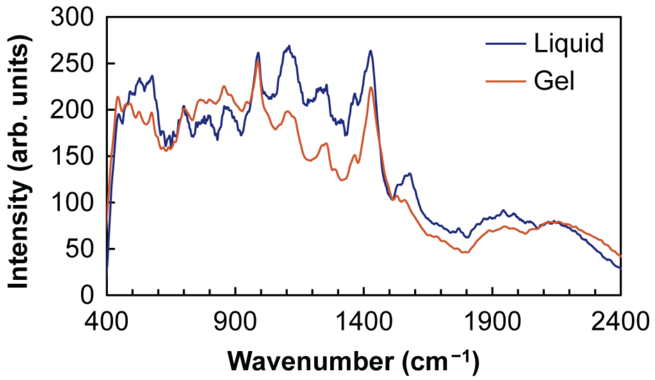

Fig S23. Variable-temperature Raman spectroscopy on 5.6 wt% PEDOT:PSS_{96}-*b*-$PNIPAM_{440}$ TR-CP. Change in intensity as a function of wavenumber, recorded at 23 °C for the liquid and 40 °C for the gel.

The bands for PEDOT, PSS, and PNIPAM are attributed based on reports in the literature,[17, 18] as summarized in **Table S5**. The intensity of bands attributed to PEDOT does not follow a particular

trend, the wavenumber remains identical in the solution and gel. Only one band is noticeable for PSS, which may demonstrate that PSS is predominantly confined to the core of the colloids. The PSS bands are likely suppressed by the more Raman-active signals for PNIPAM and PEDOT. This hypothesis, that the PSS is “hidden” due to the PNIPAM, is also supported by the low intensity of sulfur peaks in the XPS. Finally, the C-N bond vibrations arising from PNIPAM show a decrease in intensity on gelation, indicating an increase in the hydrophobic and dehydrated form of the amide bond, resulting from increased inter- and intra-molecular hydrogen bonding.

Table S5. Analysis of variable-temperature Raman Spectroscopy

Wavenumber (cm^{-1})	Change in intensity on heating (arb. units)	Band assignment
441	Increase	PEDOT, backbone
578	Decrease	PEDOT, oxyethylene ring
862.5	Increase	PEDOT, oxyethylene ring
991	No change	PEDOT, backbone and oxyethylene ring
1107	Decrease	PSS
1257	Decrease	PNIPAM, C-N with NH
1365	Decrease	PEDOT, backbone
1430	Decrease	PNIPAM
1585	Decrease, shift to 1549	PNIPAM, C-N with NH

2.8 Electronic characterization of the TR-CPs at pH = 7 in PBS

A sample of 0.6 cm^3 was used for all the measurements to facilitate comparison between the samples.

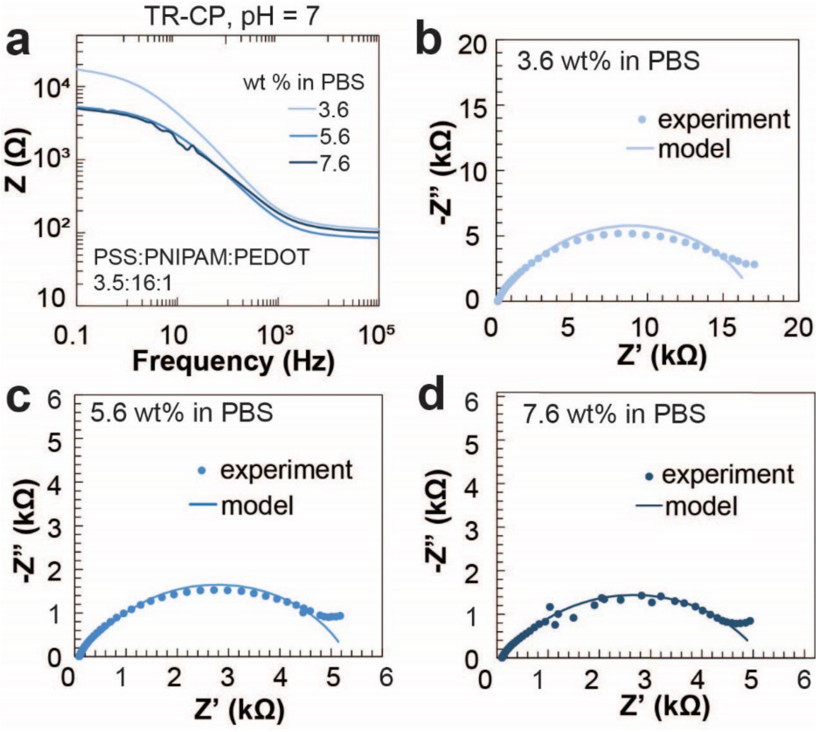

Fig S24. Electronic properties of TR-CP gel (37 °C) at different concentrations at pH 7 in PBS. **a.** Bode plot showing the change in impedance with frequency for 3.6 wt%, 5.6 wt% and 7.6 wt% TR-CP gels. Nyquist plot for TR-CP at **b.** 3.6 wt%. **c.** 5.6 wt% and **d.** 7.6 wt%.

Table S6. Fitting elements for the TR-CP at pH 7 in varying concentration in PBS by EIS.

Elements	3.6 wt% gel	5.6 wt% gel	7.6 wt% gel
R_C (Ω)	126.1	91.67	109.3
R_e (kΩ)	17.06	5.2	5.07
R_i (Ω)	1540	500.6	309
Q_{dl} ($F \cdot S^{N-1}$)	9.3×10^{-6}	1.9×10^{-5}	2.6×10^{-5}
N_{dl}	0.75	0.71	0.65
C_g (F)	1.2×10^{-6}	1.4×10^{-6}	1.1×10^{-6}
χ^2	0.025	0.016	0.011
R from 2-point probe (kΩ)	16.67	8.70	9.37

Table S7. Conductivity of TR-CPs at pH = 7 in PBS.

Sample	σ^a (mS cm^{-1})	σ_i^b (mS cm^{-1})	σ_e^b (mS cm^{-1})
3.6 wt% gel	0.099	1.082	0.097
5.6 wt% gel	0.187	3.329	0.318
7.6 wt% gel	0.177	5.393	0.329

[a]electronic conductivity calculated from 2-point probe. [b]ionic and electronic conductivity calculated from EIS.

2.9 *In vitro* cytocompatibility

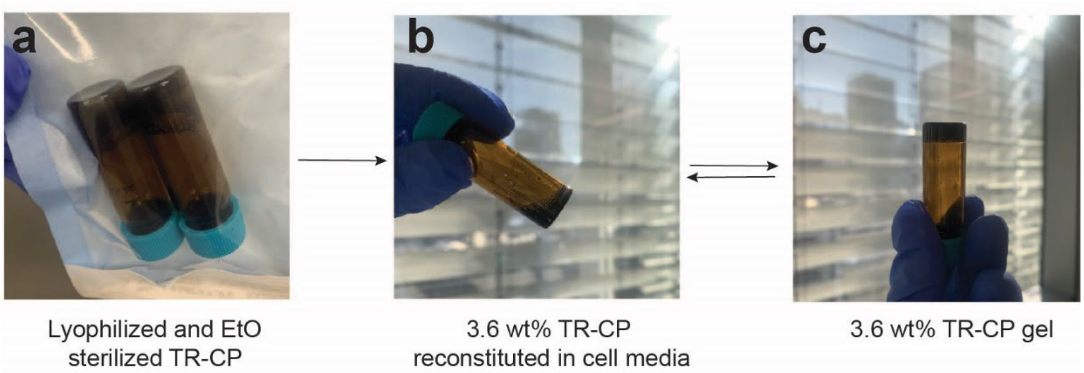

Fig. S25. Preparation of TR-CPs for cell viability experiments. a. TR-CP was neutralized, lyophilized and sterilized with ethylene oxide. b. TR-CP powder reconstituted in sterile cell media, 3.6 wt%. c. Reversible gelation of 3.6 wt% TR-CP in cell media.

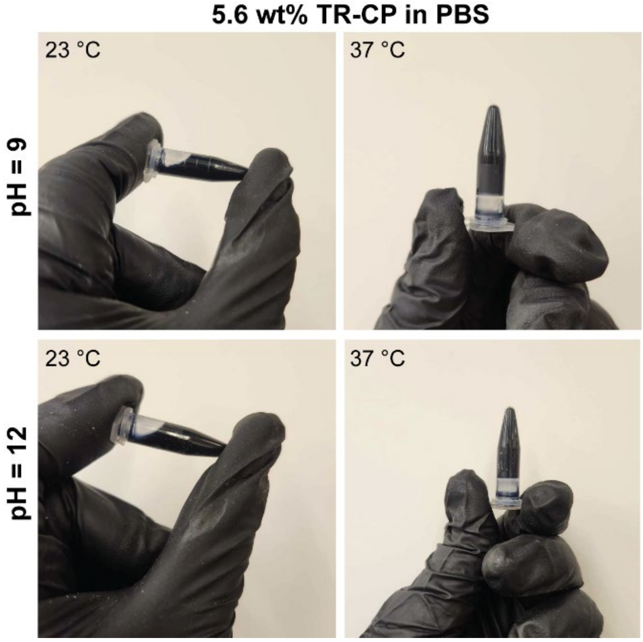

Fig. S26. Effect of basic pH on the TR-CP at pH = 9 (top) and pH = 12 (bottom). The lyophilized TR-CP powder was dispersed in PBS at 5.6 wt%, and 0.1M NaOH was added dropwise using a micropipette to adjust the pH. The TR-CP showed good dispersibility under both conditions and retained its reversible sol-gel transition. However, the TR-CP powder was not dispersible in 1M NaOH, indicating that pH = 14 was too harsh for the TR-CP.

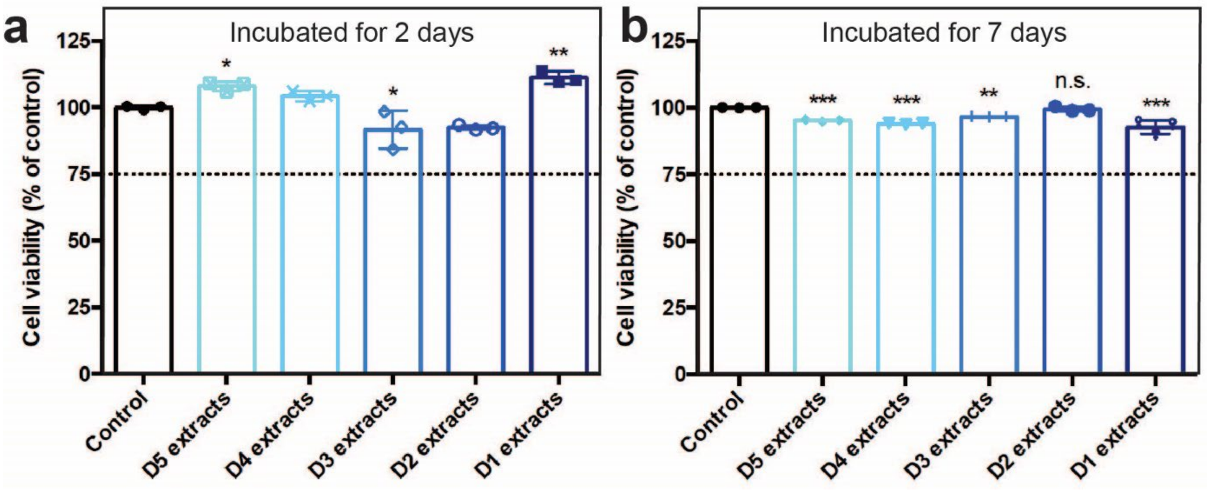

Fig. S27. Indirect contact assay for 3.6 wt% TR-CP. The alamarBlue assay of L929 cell viability after incubating with cell culture media (control) or TR-CP gel extracts of day 1-5 after **a.** 2 days and **b.** 7 days. Data presented as mean ± std. deviation of biological replicates. n = 3 for each group. Statistical significance compared against control using one-way ANOVA test; * $p < 0.05$, ** $p < 0.01$, and *** $p < 0.001$, n.s. = not significant.

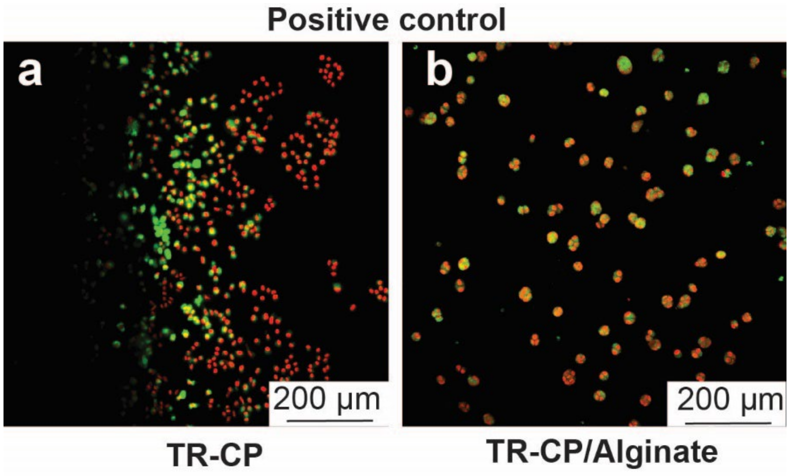

Fig. S28. Positive control for **a.** TR-CP gels: cells after incubating with control collagen gel and treated with 70% Ethanol; **b.** TR-CP/alginate composite gels: cells after incubating with control alginate gel and treated with 70% Ethanol.

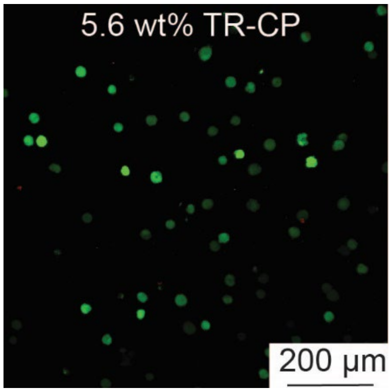

Fig S29. Cell viability for 5.6 wt% PEDOT:PSS$_{96}$-*b*-PNIPAM$_{440}$ TR-CP/alginate. Cells after incubating with 5.6% TR-CP in alginate gel.

2.10 Characterization of Alginate and TR-CP/Alginate composite gels

2.10.1 Rheological characterization.

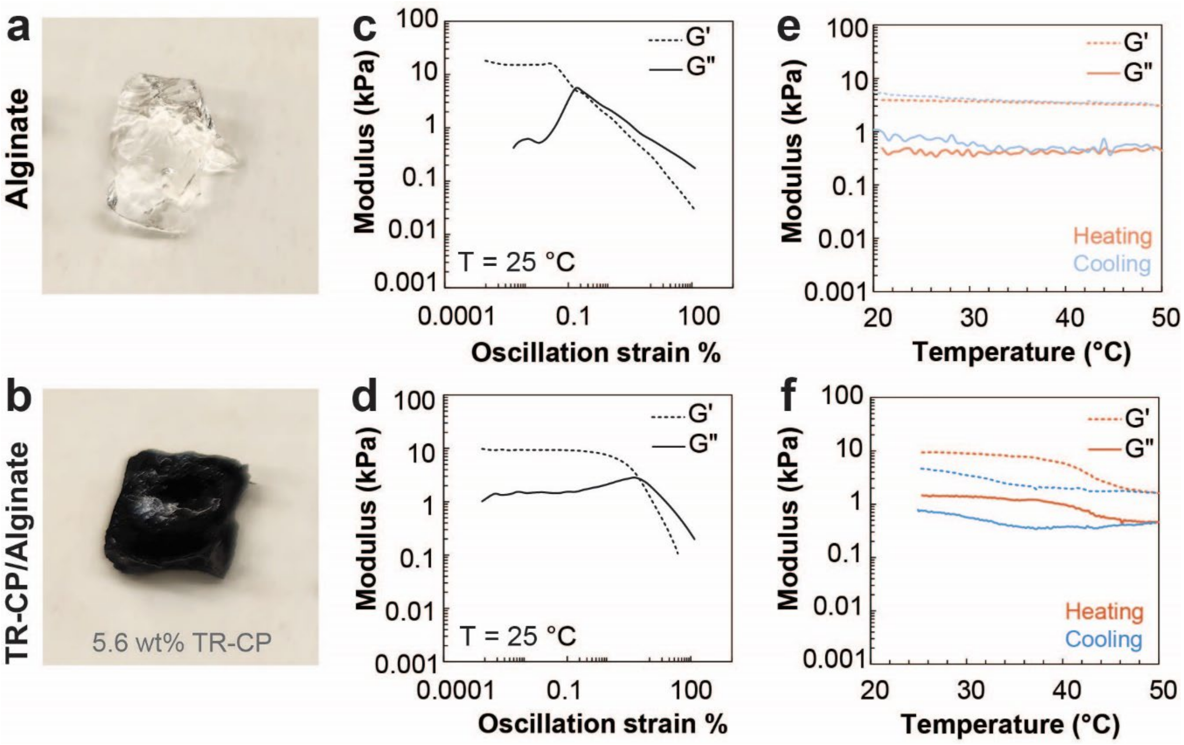

Fig S30. Rheological characterization of the alginate and 5.6 wt% TR-CP/alginate composite gels. Top row: alginate, Bottom row: TR-CP/alginate. **a.** Picture of alginate gel, made with 1 wt% solution in DI water and crosslinked using 20 mM $BaCl_2$. **b.** Picture of 5.6 wt% TR-CP/alginate gel, made with 5.6 wt% TR-CP dispersed in 1 wt% alginate solution in DI water and crosslinked using 20 mM $BaCl_2$. Change in moduli as a function of oscillation strain % for **c.** alginate and **d.** TR-CP/alginate. Change in moduli as a function of temperature for **e.** alginate and **f.** TR-CP/alginate.

2.10.2 Electronic characterization.

Measurements were taken on sample volumes of 0.6 cm^3 for both gels.

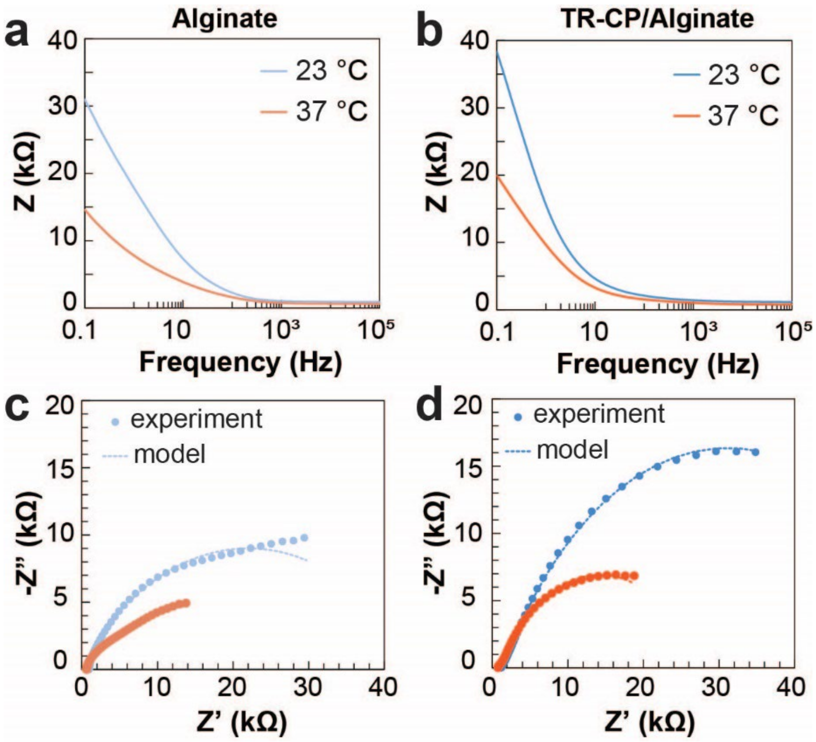

Fig S31. EIS of alginate and 5.6 wt% TR-CP/alginate gels. Bode plot (change in impedance as a function of frequency) for **a.** alginate and **b.** TR-CP/ alginate. Nyquist plots showing change in imaginary impedance vs real impedance for **c.** alginate and **d.** TR-CP/alginate.

2.11 *In vivo* cytocompatibility

Analysis. The TR-CP/alginate demonstrated decreased volume loss over the two-week period compared to the control Alginate gels as evidenced by the translucent gel layers visible in the sections. Closer inspection of the interface between the TR-CP/alginate gel and the surrounding tissue demonstrates that at 1 week, in the acute inflammatory process, the surrounding tissue is distinct from the gel, where the gel remains largely intact with minimal dissolution or cell infiltration. At 2 weeks post implantation, smaller pockets of the gel can be seen surrounded by connective tissue and cellular activity. Close inspection reveals the gels edges are being broken up slowly as the inflammatory response lessens. Additional vasculature can also be seen near the gel edges. Microvasculature formation is also seen with other conductive polymer-based scaffolds

when implanted subcutaneously.[19] This study suggests the TR-CP/Alginate gels have minimal negative effects to the overall tissue function and are able to maintain integrity longer than alginate alone.

Table S8. Description of samples and controls for cytocompatibility tests

	**Indirect contact cell viability (TR-CP)**	**Direct contact cell viability (TR-CP)**	**Direct contact cell viability (TR-CP/alginate)**	***In vivo* cytocompatibility (TR-CP/alginate)**
**Sample**	Cell media extracts from 3.6 wt% TR-CP in cell media, pH = 7-7.4	3.6 wt% TR-CP in cell media, pH = 7-7.4	TR-CP/Alginate with 3.6 wt% or 5.6 wt% loading of TR-CP	TR-CP/Alginate with 5.6 wt% loading of TR-CP
**Control**	Fresh cell media	Collagen gel	Alginate gel	Alginate gel
**Positive control**	-	Collagen gel, treated with 70% ethanol	Alginate gel, treated with 70% ethanol	-

2.12 Processing of the TR-CPs

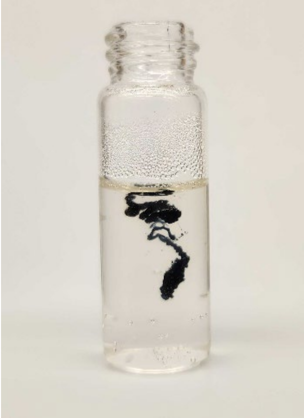

Fig. S32. Spreading of TR-CP in cooled liquid. 5.6 wt% TR-CP in PBS patterned in warm alginate and allowed to cool to room temperature (23 °C).

3 References:

(1) Yusa, S.-I.; Endo, T.; Ito, M. Synthesis of thermo-responsive 4-arm star-shaped porphyrin-centered poly(N,N-diethylacrylamide) via reversible addition-fragmentation chain transfer radical polymerization. *Journal of Polymer Science Part A: Polymer Chemistry* **2009**, *47* (24), 6827-6838. DOI: https://doi.org/10.1002/pola.23722.

(2) Mizusaki, M.; Endo, T.; Nakahata, R.; Morishima, Y.; Yusa, S.-i.; Mizusaki, M.; Endo, T.; Nakahata, R.; Morishima, Y.; Yusa, S.-i. pH-Induced Association and Dissociation of Intermolecular Complexes Formed by Hydrogen Bonding between Diblock Copolymers. *Polymers 2017, Vol. 9, Page 367* **2017**, *9* (8). DOI: 10.3390/polym9080367.

(3) Suman, K.; Wagner, N. J. Anomalous rheological aging of a model thermoreversible colloidal gel following a thermal quench. *Journal of Chemical Physics* **2022**, *157* (2), 024901-024901. DOI: 10.1063/5.0094237/2841612.

(4) Winter, H. H. Can the gel point of a cross-linking polymer be detected by the G′ – G″ crossover? *Polymer Engineering & Science* **1987**, *27* (22). DOI: 10.1002/pen.760272209.

(5) Tang, G.; Peng, L.; Baldwin, P. R.; Mann, D. S.; Jiang, W.; Rees, I.; Ludtke, S. J. EMAN2: An extensible image processing suite for electron microscopy. *Journal of Structural Biology* **2007**, *157* (1), 38-46. DOI: https://doi.org/10.1016/j.jsb.2006.05.009.

(6) Newcomb, C. J.; Moyer, T. J.; Lee, S. S.; Stupp, S. I. Advances in cryogenic transmission electron microscopy for the characterization of dynamic self-assembling nanostructures. *Current Opinion in Colloid & Interface Science* **2012**, *17* (6), 350-359. DOI: https://doi.org/10.1016/j.cocis.2012.09.004.

(7) Takano, T.; Masunaga, H.; Fujiwara, A.; Okuzaki, H.; Sasaki, T. PEDOT Nanocrystal in Highly Conductive PEDOT:PSS Polymer Films. *Macromolecules* **2012**, *45* (9), 3859-3865. DOI: 10.1021/ma300120g.

(8) Taussig, L.; Ghasemi, M.; Han, S.; Kwansa, A. L.; Li, R.; Keene, S. T.; Woodward, N.; Yingling, Y. G.; Malliaras, G. G.; Gomez, E. D.; et al. Electrostatic self-assembly yields a structurally stabilized PEDOT:PSS with efficient mixed transport and high-performance OECTs. *Matter* **2024**, *7* (3), 1071-1091. DOI: https://doi.org/10.1016/j.matt.2023.12.021.

(9) Murphy, R. J.; Weigandt, K. M.; Uhrig, D.; Alsayed, A.; Badre, C.; Hough, L.; Muthukumar, M. Scattering Studies on Poly(3,4-ethylenedioxythiophene)–Polystyrenesulfonate in the Presence

of Ionic Liquids. *Macromolecules* **2015**, *48* (24), 8989-8997. DOI: 10.1021/acs.macromol.5b02320.

(10) Pokuri, B. S. S.; Stimes, J.; O'Hara, K.; Chabinyc, M. L.; Ganapathysubramanian, B. GRATE: A framework and software for GRaph based Analysis of Transmission Electron Microscopy images of polymer films. *Computational Materials Science* **2019**, *163*, 1-10. DOI: https://doi.org/10.1016/j.commatsci.2019.02.030.

(11) Gueye, M. N.; Carella, A.; Massonnet, N.; Yvenou, E.; Brenet, S.; Faure-Vincent, J.; Pouget, S.; Rieutord, F.; Okuno, H.; Benayad, A.; et al. Structure and Dopant Engineering in PEDOT Thin Films: Practical Tools for a Dramatic Conductivity Enhancement. *Chemistry of Materials* **2016**, *28* (10), 3462-3468. DOI: 10.1021/acs.chemmater.6b01035.

(12) Wang, Y.; Zhu, C.; Pfattner, R.; Yan, H.; Jin, L.; Chen, S.; Molina-Lopez, F.; Lissel, F.; Liu, J.; Rabiah, N. I.; et al. A highly stretchable, transparent, and conductive polymer. *Science Advances* **2017**, *3* (3), e1602076. DOI: doi:10.1126/sciadv.1602076.

(13) Patel, B. B.; Pan, T.; Chang, Y.; Walsh, D. J.; Kwok, J. J.; Park, K. S.; Patel, K.; Guironnet, D.; Sing, C. E.; Diao, Y. Concentration-Driven Self-Assembly of PS-b-PLA Bottlebrush Diblock Copolymers in Solution. *ACS Polymers Au* **2022**, *2* (4), 232-244. DOI: 10.1021/ACSPOLYMERSAU.1C00057.

(14) Papagiannopoulos, A.; Zhao, J.; Zhang, G.; Pispas, S.; Radulescu, A. Thermoresponsive aggregation of PS–PNIPAM–PS triblock copolymer: A combined study of light scattering and small angle neutron scattering. *European Polymer Journal* **2014**, *56* (1), 59-68. DOI: 10.1016/J.EURPOLYMJ.2014.04.013.

(15) Pesek, S. L.; Xiang, Q.; Hammouda, B.; Verduzco, R. Small-angle neutron scattering analysis of bottlebrush backbone and side chain flexibility. *Journal of Polymer Science Part B: Polymer Physics* **2017**, *55* (1), 104-111. DOI: 10.1002/POLB.24251.

(16) Kotlarchyk, M.; Chen, S. H. Analysis of small angle neutron scattering spectra from polydisperse interacting colloids. *The Journal of Chemical Physics* **1983**, *79* (5), 2461-2469. DOI: 10.1063/1.446055 (acccessed 5/11/2025).

(17) Ahmed, Z.; Gooding, E. A.; Pimenov, K. V.; Wang, L.; Asher*, S. A. UV Resonance Raman Determination of Molecular Mechanism of Poly(<i>N</i>-isopropylacrylamide) Volume Phase Transition. *The Journal of Physical Chemistry B* **2009**, *113* (13). DOI: 10.1021/jp810685g.

(18) Kong, M.; Garriga, M.; Reparaz, J. S.; Alonso, M. I. Advanced Optical Characterization of PEDOT:PSS by Combining Spectroscopic Ellipsometry and Raman Scattering. *ACS Omega* **2022**, *7* (43), 39429-39436. DOI: doi.org/10.1021/acsomega.2c05945.

(19) Sun, K. H.; Liu, Z.; Liu, C. C.; Yu, T.; Shang, T.; Huang, C.; Zhou, M.; Liu, C. C.; Ran, F.; Li, Y.; et al. Evaluation of in vitro and in vivo biocompatibility of a myo-inositol hexakisphosphate gelated polyaniline hydrogel in a rat model. *Scientific Reports* **2016**, *6* (1), 23931-23931. DOI: 10.1038/srep23931.
