# Supplementary material for: Thermo-reversible gelation of self-assembled conducting polymer colloids
Source: Nat Commun. 2025 Dec 5;16:10879. doi: 10.1038/s41467-025-66034-x (PMC12680621; doi:10.1038/s41467-025-66034-x)
Supplement: Supplementary file 2 — Description of Additional Supplementary Files [file 41467_2025_66034_MOESM2_ESM.pdf]

Description of Additional Supplementary Files

File Name: Supplementary Movie 1
Description: Video showing the reversible sol-gel transition of the TR-CP.

File Name: Supplementary Movie 2
Description: Video demonstrating the self-healing behavior of the TR-CP.

File Name: Supplementary Movie 3
Description: Video showing the injection of the TR-CP into a warm alginate solution.

File Name: Supplementary Movie 4
Description: Video of the setup and recording of muscle activity (fist closing) by surface electromyography using TR-CP electrodes.
