# Supplementary material for: Thermo-reversible gelation of self-assembled conducting polymer colloids
Source: Nat Commun. 2025 Dec 5;16:10879. doi: 10.1038/s41467-025-66034-x (PMC12680621; doi:10.1038/s41467-025-66034-x)
Supplement: Supplementary file 7 — Reporting Summary [file 41467_2025_66034_MOESM7_ESM.pdf]

nature portfolio

Corresponding author(s): Laure V. Kayser

Last updated by author(s): 10/28/2025

Reporting Summary

Nature Portfolio wishes to improve the reproducibility of the work that we publish. This form provides structure for consistency and transparency in reporting. For further information on Nature Portfolio policies, see our Editorial Policies and the Editorial Policy Checklist.

Statistics

For all statistical analyses, confirm that the following items are present in the figure legend, table legend, main text, or Methods section.

n/a	Confirmed	
☐	☒	The exact sample size (*n*) for each experimental group/condition, given as a discrete number and unit of measurement
☐	☒	A statement on whether measurements were taken from distinct samples or whether the same sample was measured repeatedly
☐	☒	The statistical test(s) used AND whether they are one- or two-sided<br>*Only common tests should be described solely by name; describe more complex techniques in the Methods section.*
☒	☐	A description of all covariates tested
☒	☐	A description of any assumptions or corrections, such as tests of normality and adjustment for multiple comparisons
☐	☒	A full description of the statistical parameters including central tendency (e.g. means) or other basic estimates (e.g. regression coefficient) AND variation (e.g. standard deviation) or associated estimates of uncertainty (e.g. confidence intervals)
☐	☒	For null hypothesis testing, the test statistic (e.g. *F*, *t*, *r*) with confidence intervals, effect sizes, degrees of freedom and *P* value noted<br>*Give P values as exact values whenever suitable.*
☒	☐	For Bayesian analysis, information on the choice of priors and Markov chain Monte Carlo settings
☒	☐	For hierarchical and complex designs, identification of the appropriate level for tests and full reporting of outcomes
☒	☐	Estimates of effect sizes (e.g. Cohen's *d*, Pearson's *r*), indicating how they were calculated

Our web collection on statistics for biologists contains articles on many of the points above.

Software and code

Policy information about availability of computer code

Data collection	No commercial, open source or custom codes were used.
Data analysis	MestreNova was used for NMR analysis. TRIOS was used to extract rheology data. NOVA 2.5 was used to extract EIS data. DigitalMicrograph software was used for the analysis of cryo-EM micrographs, including line scan and Fast Fourier Transform (FFT) analysis. Fiji software and radial integration plugin were used for radial integration of 2D FFT plots. For small angle X-ray scattering, background subtraction and averaging of the data were performed using JupyterHub, which was provided by Brookhaven National Laboratory. X-ray photoelectron Spectra of the samples were analyzed using Thermo Scientific Avantage Data System software. ImageJ was used to analyze cell images and tissue sections.

For manuscripts utilizing custom algorithms or software that are central to the research but not yet described in published literature, software must be made available to editors and reviewers. We strongly encourage code deposition in a community repository (e.g. GitHub). See the Nature Portfolio guidelines for submitting code & software for further information.

Data

Policy information about availability of data

All manuscripts must include a data availability statement. This statement should provide the following information, where applicable:

- Accession codes, unique identifiers, or web links for publicly available datasets
- A description of any restrictions on data availability
- For clinical datasets or third party data, please ensure that the statement adheres to our policy

The source data for NMR, GPC, rheology, EIS, SAXS, XPS, Raman spectroscopy generated in this study have been deposited in the Figshare database under accession code https://doi.org/10.6084/m9.figshare.29260157. All other data generated and analyzed in this study are provided in the Supplementary Information file: synthesis and materials preparation, molecular characterization: NMR and GPC for PSS CTAs and PSS-b-PNIPAM, Experimental design, rheological characterization of TR-CP, effect of TR-CP concentration. Stability of TR-CP, electronic characterization of TR-CP, TR-CP microstructure characterization using cryo-EM, rheology, SAXS, XPS, and Raman spectroscopy, electronic characterization of TR-CP at pH = 7, in vitro cytocompatibility, characterization of TR-CP/alginate, in vivo cytocompatibility and subcutaneous implantation of TR-CP/alginate gels, processing of TR-CP, and surface EMG measurements.

Research involving human participants, their data, or biological material

Policy information about studies with human participants or human data. See also policy information about sex, gender (identity/presentation), and sexual orientation and race, ethnicity and racism.

Reporting on sex and gender	N.A.
Reporting on race, ethnicity, or other socially relevant groupings	N.A.
Population characteristics	N.A.
Recruitment	N.A.
Ethics oversight	The participant in Fig. 6 provided written, informed consent.

Note that full information on the approval of the study protocol must also be provided in the manuscript.

Field-specific reporting

Please select the one below that is the best fit for your research. If you are not sure, read the appropriate sections before making your selection.

- [x] Life sciences
- [ ] Behavioural & social sciences
- [ ] Ecological, evolutionary & environmental sciences

For a reference copy of the document with all sections, see nature.com/documents/nr-reporting-summary-flat.pdf

Life sciences study design

All studies must disclose on these points even when the disclosure is negative.

Sample size	For in vitro studies n=3 biological replicates were used per samples to account for inherent biological variability between different cell cultures. For in vivo studies, 2 animals were used, 1 per time point, with n=2 biological replicates per animal. Collected data was merely qualitative, thus a small sample size was sufficient.
Data exclusions	No data was excluded from the in vitro or in vivo experiments.
Replication	For in vitro studies, 3 biological replicates were performed. In vivo studies were not repeated but within each animal, 2 biological replicates were performed. Replication was successful
Randomization	For in vitro studies, different experimental conditions were randomly assigned to the various cell samples being tested. For in vivo studies, samples were randomly placed within animal models for implantation.
Blinding	For in vitro studies, no blinding was performed because color difference between control and experimental groups. For in vivo studies, implantation, explantation, and staining were performed blinded.

Reporting for specific materials, systems and methods

We require information from authors about some types of materials, experimental systems and methods used in many studies. Here, indicate whether each material, system or method listed is relevant to your study. If you are not sure if a list item applies to your research, read the appropriate section before selecting a response.

Materials & experimental systems

n/a	Involved in the study	
☒	☐	Antibodies
☐	☒	Eukaryotic cell lines
☒	☐	Palaeontology and archaeology
☐	☒	Animals and other organisms
☒	☐	Clinical data
☒	☐	Dual use research of concern
☒	☐	Plants

Methods

n/a	Involved in the study	
☒	☐	ChIP-seq
☒	☐	Flow cytometry
☒	☐	MRI-based neuroimaging

Eukaryotic cell lines

Policy information about cell lines and Sex and Gender in Research

Cell line source(s)	L929 Fibroblasts from ATCC derived from normal subcutaneous areolar and adipose tissue of a 100-day-old male C3H/An mouse
Authentication	No cell line authentication was performed
Mycoplasma contamination	Cell line was tested for mycoplasma contamination before receipt by lab.
Commonly misidentified lines (See ICLAC register)	No commonly misidentified lines were used in this study

Animals and other research organisms

Policy information about studies involving animals; ARRIVE guidelines recommended for reporting animal research, and Sex and Gender in Research

Laboratory animals	Male Sprague Dawley rats (3 months old) were ordered from Charles River Laboratories
Wild animals	No wild animals were used
Reporting on sex	Only male rats were used. Male rats offer a relatively inexpensive and widely accepted model for subcutaneous implantation. Sex should not impact greatly subcutaneous response to the material in question.
Field-collected samples	No field-collected samples were used
Ethics oversight	Animal protocol was overseen and approved by Northwestern University's Institutional Animal Care and Use Committee with approval number IS00024034.

Note that full information on the approval of the study protocol must also be provided in the manuscript.

Plants

Seed stocks	No plants were used
Novel plant genotypes	No plants were used
Authentication	No plants were used
